# Identification of Adaptive Mutations in the Influenza A Virus Non-Structural 1 Gene That Increase Cytoplasmic Localization and Differentially Regulate Host Gene Expression

**DOI:** 10.1371/journal.pone.0084673

**Published:** 2013-12-31

**Authors:** Nicole Forbes, Mohammed Selman, Martin Pelchat, Jian Jun Jia, Alain Stintzi, Earl G. Brown

**Affiliations:** 1 Department of Biochemistry, Microbiology and Immunology, Faculty of Medicine, University of Ottawa, Ottawa, Ontario, Canada; 2 Emerging Pathogens Research Centre, Faculty of Medicine, University of Ottawa, Ottawa, Ontario, Canada; University of Edinburgh, United Kingdom

## Abstract

The NS1 protein of influenza A virus (IAV) is a multifunctional virulence factor. We have previously characterized gain-of-function mutations in the NS1 protein arising from the experimental adaptation of the human isolate A/Hong Kong/1/1968(H3N2) (HK) to the mouse. The majority of these mouse adapted NS1 mutations were demonstrated to increase virulence, viral fitness, and interferon antagonism, but differ in binding to the post-transcriptional processing factor cleavage and polyadenylation specificity factor 30 (CPSF30). Because nuclear trafficking is a major genetic determinant of influenza virus host adaptation, we assessed subcellular localization and host gene expression of NS1 adaptive mutations. Recombinant HK viruses with adaptive mutations in the NS1 gene were assessed for NS1 protein subcellular localization in mouse and human cells using confocal microscopy and cellular fractionation. In human cells the HK wild-type (HK-wt) virus NS1 protein partitioned equivalently between the cytoplasm and nucleus but was defective in cytoplasmic localization in mouse cells. Several adaptive mutations increased the proportion of NS1 in the cytoplasm of mouse cells with the greatest effects for mutations M106I and D125G. The host gene expression profile of the adaptive mutants was determined by microarray analysis of infected mouse cells to show either high or low extents of host-gene regulation (HGR or LGR) phenotypes. While host genes were predominantly down regulated for the HGR group of mutants (D2N, V23A, F103L, M106I+L98S, L98S, M106V, and M106V+M124I), the LGR phenotype mutants (D125G, M106I, V180A, V226I, and R227K) were characterized by a predominant up regulation of host genes. CPSF30 binding affinity of NS1 mutants did not predict effects on host gene expression. To our knowledge this is the first report of roles of adaptive NS1 mutations that impact intracellular localization and regulation of host gene expression.

## Introduction

The influenza A virus (IAV) NS1 protein possesses multiple functions that support virus replication. NS1 can engage in many functions due to its ability to translocate to both the nucleus and cytoplasm of infected cells and interact with numerous cellular and viral factors including RNA. Cytoplasmic activities include blocking viral RNA detection by RIG-I signalling of type I interferon (IFN) induction as well as inhibition of IFN-stimulated antiviral proteins, suppression of the host cell apoptotic response, and enhancement of viral protein synthesis [1]. In the nucleus, NS1 binds cellular post-transcriptional processing factors including the cellular post-transcriptional host factor cleavage and polyadenylation specificity factor 30 (CPSF30), which has been reported to result in a blockade of host gene expression, including type I IFN [2], [3].

NS1 localization is governed by two known nuclear localization signals (NLS) (NLS1: aa 34–38, NLS2: aa 203–237), which interact with the cellular protein importin α [4] and induce rapid nuclear localization following translation [5]. Later in infection, the protein is detected in both the nucleus and the cytoplasm [6], which is attributed to interaction of its NES element (137–147) [6] with the nuclear pore complex. Cytoplasmic NS1 levels are also influenced by an inhibitory sequence (148–161) adjacent to the NES [7]. NS1 also localizes to the nucleolus and contains a NoLS involving key basic residues Arg-224 and Arg-229 [4], [8], however the role of NS1 nucleolar localization and its contribution to viral replication is unknown.

The ability of the NS1 protein to translocate within discrete cellular compartments and engage in pro-viral functions including blockade of host gene expression and type I interferon (IFN) induction has been shown to be essential for optimum virus replication. Alanine substitutions at NLS residues 38 and 41 were observed to reduce NS1 nuclear localization and the ability to bind RNA, resulting in increased IFNα/β production and attenuation of virus replication [9], [10]. Li *et al.* (2011) showed alanine substitutions at Leu-69 and Leu-77 within the highly conserved NS1 linker region induced *de novo* localization to the nucleolus, and dramatically reduced the ability of NS1 to limit IFN induction *in vitro* [11]. In addition, NS1 binding affinity for nuclear and nucleolar factors, including CPSF30, nucleolin, and fibrillarin, also influence protein’s sequestration in the nucleus and nucleolus, respectively [8], [12]. In the case of CPSF30, amino acid mutations F103S and M106I disrupted CPSF30 binding while shifting NS1 localization to the cytoplasm [12].

Although intracellular localization of the NS1 protein is well documented, the effect of virus adaptation to a novel host on NS1 protein localization remains unknown. Cellular distribution of the NS1 protein is dependent upon virus-host interactions; several reports have shown strain-specific and cell-line specific localization patterns of the NS1 protein [4], [12], [13]. Moreover, the nuclear import signals of influenza RNP (polymerase subunits (PB1, PB2, and PA) and NP) have been shown to be determinants of adaptive evolution in mice [14]–[17] and for highly pathogenic avian influenza in mammalian cells (reviewed in [18]). We hypothesize that subcellular trafficking of NS1 is a determinant of host adaptation.

The influenza A virus host range is extensive; while aquatic waterfowl species define the primary viral reservoir, many avian and mammalian species are also hosts. However, the genetic determinants of IAV host range and virulence remain largely unknown. To this end, we previously reported the experimental evolution of the human influenza A virus isolate A/Hong Kong/1/1968 (H3N2) (HK) to high virulence in the mouse [15], [17] and identified multiple mouse-adapted (MA) mutations in the PB2 [16], [17], HA [16], [17], [19] as well as the NS1 protein [20], [21]. These mutations, including 11 in the NS1 gene, were confirmed to mediate host adaptation, assessed by enhanced virus replication in mouse lungs and/or mouse cells [21]. These mutants possessed additional adaptive phenotypes, including enhanced viral gene expression (at both transcriptional and translational levels) as well as an ability to reduce IFN-β induction in mouse lungs. Remarkably, 8 mutants lost the ability to bind CPSF30 while 4 maintained binding [21], and this trait did not correlate with IFN-β production *in vivo* in the mouse lung. One of the NS1 mutants, D125G, also induced expression of a novel viral protein, the non-structural 3 protein (NS3), due to activation of an alternative splice site [21], [22]. Although the function of this novel protein is currently under investigation, we have confirmed that expression of the NS3 protein enhances viral replication for the recombinant HK virus expressing the NS1 D125G mutation [22].

The adaptive phenotypes associated with MA NS1 mutants were thus wide ranging, but more importantly, involved NS1 protein functions occurring in both the nucleus and the cytoplasm of infected cells. As the HK virus is a human virus isolate, we hypothesized that its evolution to high virulence in the mouse selected for mutations in the NS1 protein that would favour NS1 cellular localization optimal for replicative fitness in the mouse. Furthermore several human HK NS1 mutations selected on mouse adaptation mapped to regions within or near to known NLS and NES domains. We next sought to determine the effect of MA NS1 mutations on NS1 localization in mouse and human infected cells, and whether altered NS1 localization correlated with IAV adaptation to a novel host. To further understand the role of CPSF30 binding during infection, we also assessed whether CPSF30 binding affinity of the adaptive mutants correlated with altered host gene expression in mouse cells.

We found that the MA NS1 mutant proteins were present at a significantly higher proportion in the cytoplasm of infected mouse cells compared to wild-type HK NS1, and that NS1 mutants altered NS1 cellular localization in a host cell-dependent manner. We previously have shown that the majority of MA NS1 mutations abrogated binding to the CPSF30 protein [21]; here we demonstrate two distinct phenotypes of altered host gene expression with either high or low effects upon infection of mouse cells with NS1 mutant HK viruses, and moreover, that CPSF30 binding affinity did not correlate with reduced expression of host genes. To our knowledge, this study is the first to identify the roles for NS1 localization in host adaptation as well as in regulating general host gene expression.

## Materials and Methods

### Cell Lines

MDCK cells (Madin-Darby canine kidney; Health Canada), M1 cells (Mouse kidney epithelium; ATCC), and A549 cells (human lung carcinoma; ATCC) were maintained in minimum essential medium (MEM). MEM was supplemented with L-glutamine (2 mM), Penicillin (100 U/ml), Streptomycin (100 ug/ml) and fetal bovine serum (FBS) (10%). All cells were incubated at 37°C in the presence of 5% CO_2_.

### Viruses

The H3N2 human influenza isolate A/Hong Kong/1/1968 (HK-wt) was originally obtained from the Laboratory Center for Disease Control (Health Canada, Ottawa, Canada). The H1N1 human influenza isolate A/Puerto Rico/8/34 (PR8) was obtained by reverse genetics of the 8 viral genome segments expressed on pHW2000 plasmids gratefully obtained from R. Webby (St. Jude’s Children’s Research Hospital, Memphis). The laboratory adapted H1N1 influenza isolate A/WSN/33 (WSN) was obtained by reverse genetics of the 8 viral genome segments in pHH21 plasmids obtained from Yoshihiro Kawaoka that were subcloned into the pLLB plasmids for virus rescue (University of Madison, Wisconsin). Viruses were plaque purified on MDCK cells and grown in the allantoic cavity of 10-day-old specific pathogen free (SPF) chicken embryos (Canadian Food Inspection Agency, Ottawa).

### Mouse Adaptation of A/Hong Kong/1/1968 (H3N2)

The generation of mouse-adapted (MA) variants of A/Hong Kong/1/1968 (H3N2) was described previously [15], [17]. Briefly, highly virulent variants were selected after 20 or 21 passages (MA20 or MA21) in the CD-1 mouse lung. Following final passage, lung tissue was homogenized then serially plaque purified on MDCK cells.

### Generation of Recombinant NS Mutant Hong Kong viruses

To determine the role of each mutant MA NS1 gene in virulence, recombinant HK viruses were generated to express the mutations of each mouse-adapted NS gene segment (rHK NS mutants) as previously described [21]. The NS gene segments of all rescued viruses were sequenced to verify that they were free of unwanted mutations.

### Confocal Microscopy

Localization pattern of NS1 in infected cells were observed by seeding M1 or A549 cells to 60–80% confluency on 8 chamber immunoslides then infecting the cells with rHK NS1 mutants or HK-wt virus, as well as the prototype mouse-adapted viruses PR8 and WSN, at an MOI = 3 in the absence of trypsin. At 16 hpi, the cells were washed with 1× PBS (PBS), permeabilized with 0.1% Triton X100 in PBS for 5 minutes, then washed with PBS and fixed in 3.7% formaldehyde for 10 minutes. Following two washes in PBS, the cells were incubated with rabbit anti-HK NS1 polyclonal antibody, rabbit anti-HK NP polyclonal antibody, or mouse anti-NS1 monoclonal antibody overnight at 4°C. All antibodies were previously pre-adsorbed in mouse lung extract to reduce non-specific cross-reactivity and suspended in PBS washing buffer containing 3% bovine serum albumin and 0.3% Triton X-100. The cells were washed twice with PBS washing buffer then incubated with secondary Cy3-conjugated donkey anti-rabbit or donkey anti-mouse IgG, respectively (Jackson ImmunoResearch laboratories Inc., ME), for 2 hours at room temperature. Following two washes with PBS, the nuclei were labeled by DAPI incubation in the dark for 15 minutes (300 ng/mL). Excess stain was removed by 3 washes with dH_2_O. Cover slides were mounted with Dako mounting medium (Dako, Denmark) as per the manufacturer’s protocol. The cells were viewed using a Zeiss LSM 510 META/AxioVert 200 Confocal Microscope 63× oil immersion objective lens. Representative images were processed in parallel (GNU Image Manipulation Program 2.0).

### Quantification of NS1 in Cellular Compartments of Infected Cells

Confluent monolayers of M1 or A549 cells were infected (n = 2–3) with rHK NS1 mutant or HK-wt viruses as well as the prototype MA viruses A/PR/8/34 and A/WSN/33 (MOI = 2) in the absence of trypsin. At 16 hpi, the cells were washed once with ice cold 1× PBS then lysed with ice cold 1% NP-40 (in PBS) and scraped to collect adherent cells. Cells were fractioned as previously described [23], [24]. Briefly, an aliquot of the cell lysate was obtained to represent the whole cell lysate, and the remainder was centrifuged for 2 minutes at 13.5 K RPM. The supernatant containing the cell cytoplasm was then collected. The nuclear pellet (that employed twice as many cells as the W and C samples to counteract losses in preparation) was further washed with NP-40 lysis buffer and re-centrifuged. The supernatant was discarded and the pellet was re-suspended in 50 uL NP-40 lysis buffer. Whole cell, cytoplasmic, and nuclear fractions were then mixed with 4× Laemmli sample buffer and denatured by 3 minutes at 90°C prior to protein separation by SDS-PAGE gel electrophoresis. Immunoblots were probed for NS1 (polyclonal rabbit anti-NS1), histone H3 (rabbit anti-histone H3, CT, pan clone A35; Millipore, Billerica, MA, USA) and β-tubulin (mouse anti-tubulin α; Sigma, Burlington, ON). Antibodies were detected by incubation with HRP conjugated goat anti-rabbit or goat anti-mouse antibody (Sigma, Burlington, ON) followed by SuperSignal West Pico chemiluminescent substrate (Pierce, Thermo Fisher Scientific, Nepean, ON) and exposure to film.

### Densitometry Analysis

Band density of proteins detected by Western blot was quantified using the UN-SCAN-IT software (Scientific Software Solutions). As the NS1 bands associated with HK-wt and M106V+M124I infections were much weaker than the remainder of NS1 bands, relative NS1 band density was determined using two exposures (a longer exposure to quantify HK-wt and M106V +M124I NS1 bands, shown in each figure, and a shorter exposure that allowed the remainder of NS1 bands to remain below oversaturation (data not shown)). To determine the cellular distribution of the NS1 protein in infected cells, we determined the ratio of cytoplasmic NS1 to nuclear NS1. As the histone H3 band was inconsistent between the whole cell and nuclear fractions (possibly due to losses from the washing of nuclei by sedimentation involved in the fractionation procedure), the amount of NS1 protein in the nuclear and cytoplasmic fractions of a cell infected with a given virus was normalized to histone H3 and tubulin levels of the whole cell lysate, respectively. The fraction of cytoplasmic to nuclear NS1 protein was calculated by formula; (histone W/histoneN)×NS1 N = normalized NS1 N to normalize nuclear NS1 quantities to W fraction, as well as for cytoplasmic fractions (tubulin W/tubulin C)×NS1 C = normalized NS1 C, to obtain values to determine the ratio of C/N (using both values as normalized to W).

### Microarray Analysis

Confluent monolayers of M1 cells in 35 mm dishes were infected with HK-wt or rHK NS mutant viruses (MOI = 2) or mock-infected with PBS in triplicate for n = 3 biological replicates as per the previous infection protocols. At 8 hpi, the cellular RNA was collected using manufacturer’s protocol (QIAGEN RNeasy mini-kit) quantified using a PowerWave XS2, Microplate Spectrophotometer (BioTek Instruments, Inc. Winooski, VT United States ), and ribosomal RNA quality and integrity was confirmed using an Agilent 2100 Bioanalyzer (Agilent Technologies Canada Inc. Mississauga, Canada). The microarray analysis was performed by StemCore Laboratories (Ottawa, Ontario, Canada) using Genechip Mouse Exon 1.0 ST Array (Affymetrix, Santa Clara, CA, USA). Microarray gene expression data were analyzed by Flexarray1.6.1 after normalization using Affymetrix Power Tools (AFT) [25]. Genes were considered as up or down regulated relative to mock infected cells if they were ≤1 or ≥1 log2 fold different (≤ or ≥2 fold differences) expression level and were statistically significant at the p≤0.05 level using ANOVA. Genes were included for hierarchical clustering analysis among mutants if they were differentially regulated (≤ or ≥2 fold differences) and significantly different from mock-infected cells for one or more of the mutants. Microarray data were further analyzed using Bioconductor software packages in R for clustered heat maps of gene expression and mutants, Pheatmaps, (pheatmap_0.7.4.tar.gz) analysis using the complete linkage and Euclidian distance method; and gene ontology, GoProfiles (goProfiles_1.20.0.tar.gz), (available at http://bioconductor.org/biocLite.R) with outputs plotted with gplots_2.11.0.tgz and user generated software. The microarray data have been deposited in NCBI’s Gene Expression Omnibus and are accessible through GEO Series accession number GSE48217 (http://www.ncbi.nlm.nih.gov/geo/query/acc.cgi?acc=GSE48217).

### Statistical Analysis

Unless otherwise indicated, statistical significance was measured using the student t-test (Microsoft Excel, 2007) using the parameters of equal variance and two-tailed significance with a probability of <0.05 considered as statistically significant. Values were calculated as means ± standard deviation (SD), unless otherwise noted as means ± standard error (SE).

## Results

### NS1 Mutants Alter Subcellular Localization of the NS1 Protein in Infected Mouse Cells

We have previously shown that NS1 mutations selected upon mouse adaptation of the A/Hong Kong/1/1968 H3N2 virus (MA NS1 mutations) are adaptive, and associated with increased virulence in the mouse, decreased IFNβ production in the mouse lung, and increased viral replication, both *in vivo* and *in vitro,* of which the latter was associated with enhanced viral gene expression at the level of viral mRNA and protein synthesis [21]. We next assessed whether virus adaptation to a novel host alters NS1 localization using confocal microscopy. Mouse M1 cells were infected with recombinant HK viruses expressing MA NS1 mutations (rHK NS1 mutants) at a MOI of 3 followed by formaldehyde fixation at 16 hours post infection (hpi) and immunostaining using an anti-NS1 or an anti-NP (for virus protein control) polyclonal antibody with a Cy3- conjugated secondary antibody, and DAPI staining to detect the nucleus with images obtained by confocal microscopy using a 63× oil immersion objective. Given that mouse adapted NS1 mutants were previously shown to differentially increase viral fitness and viral protein production in the mouse [21], we saw similar or increased intensity of NP staining for each mutant relative to HK-wt (Figure S1).

**Figure 1:**
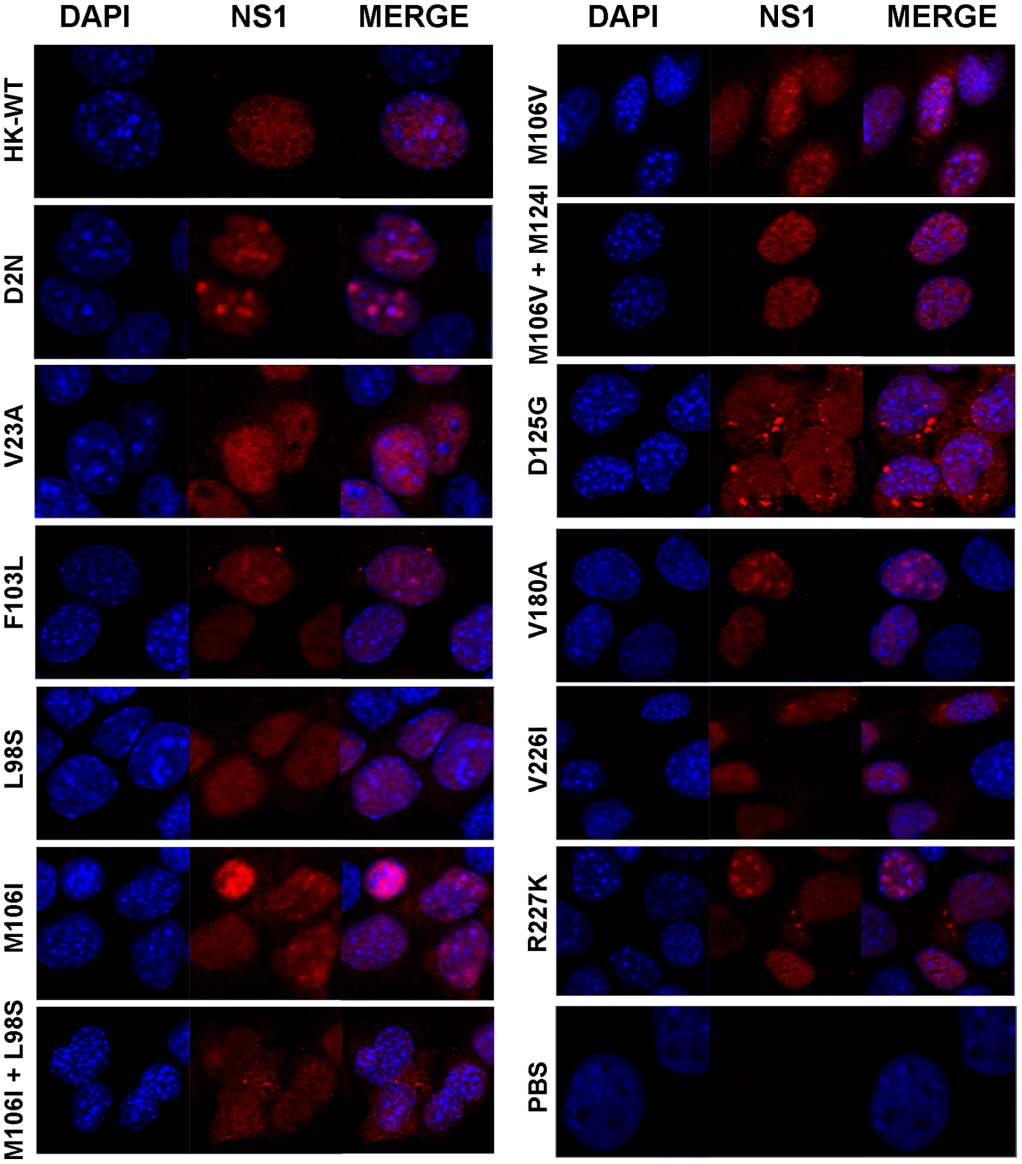
MA NS1 mutants alter subcellular localization of the NS1 protein in infected mouse cells. Mouse M1 cells were infected at MOI = 3 with rHK NS mutant viruses or the HK-wt virus, or mock infected with PBS. Following 16 hpi, cells were fixed and stained using a polyclonal anti-NS1 primary antibody and Cy3-conjugated secondary antibody as well as DAPI to localize the nucleus. Representative images are shown (taken using a 63× oil immersion objective).

The majority of mouse cells that were infected with the HK-wt virus demonstrated staining for the NS1 protein exclusively in the nucleus (Figure 1), with only 6% of cells positive for NS1 in both cellular compartments (Figure S2). However, mouse cells infected with NS1 mutants D125G and M106I+L98S, and to a lesser extent M106I and V226I, significantly increased the observed frequency of NS1 staining within the cytoplasm to up to 64% (p<0.001; D125G) (Figure 1 and S2). Thus several NS1 adaptive mutations increased cytoplasmic localization of the NS1 protein in mouse cells detectable by microscopy.

### NS1 Mutants Temporally Increase Localization of the NS1 Protein to the Cytoplasm in Infected Mouse Cells

**Figure 2:**
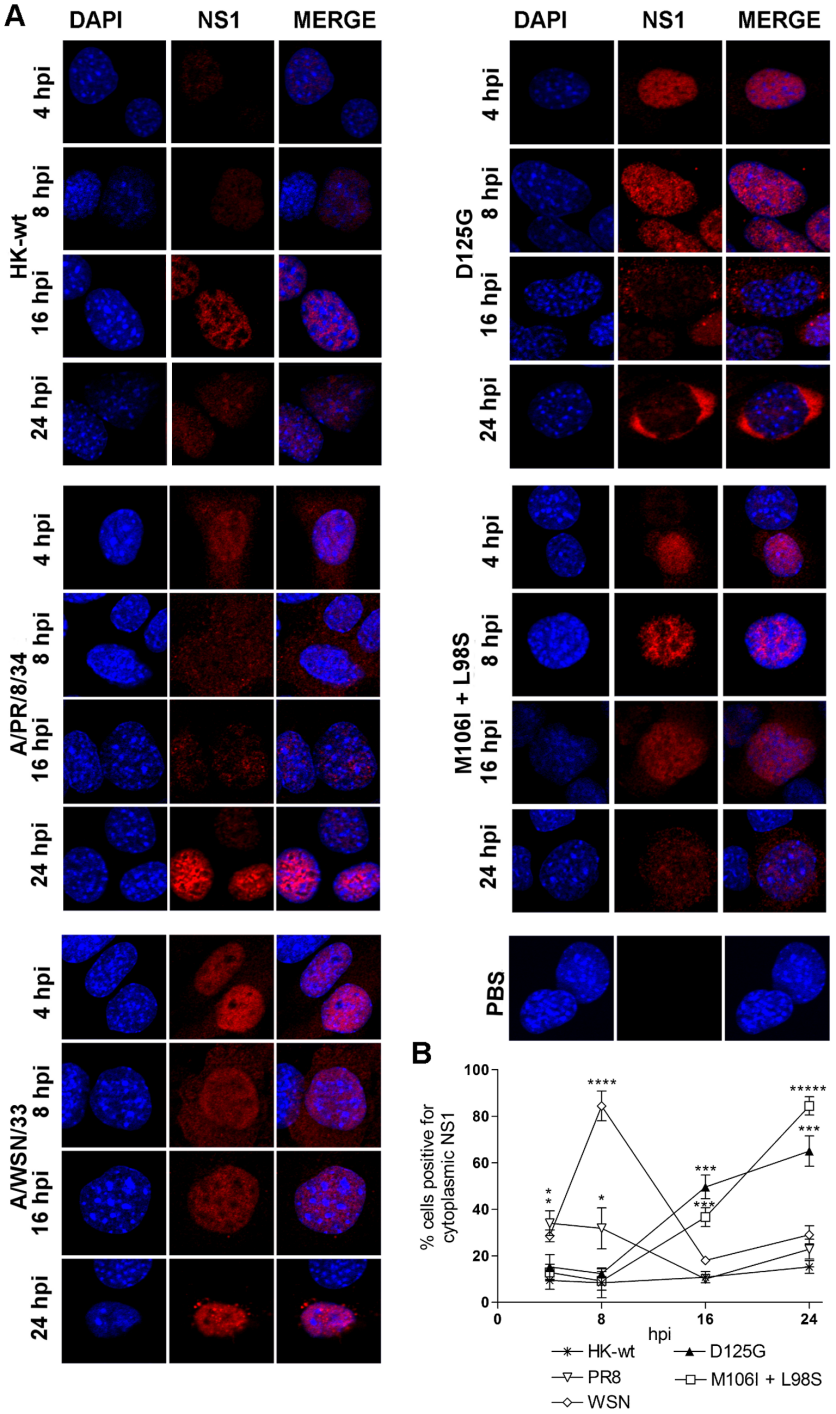
MA NS1 mutants M106I+L98S and D125G temporally increase cytoplasmic NS1 localization in infected mouse cells. Mouse M1 cells were infected at MOI = 3 with the rHK NS mutant viruses, the HK-wt virus, or prototype mouse-adapted viruses A/PR/8/34 (H1N1) (PR8) or A/WSN/33 (H1N1) (WSN). Following 4, 8, 16, and 24 hpi, cells were fixed and stained using a polyclonal anti-NS1 antibody and Cy3-conjugated secondary as well as DAPI to localize the nucleus. (A) Representative images are shown, taken at 63× using oil immersion. (B) Data represent the average percentage of cells positive for the NS1 antigen detected in the cytoplasm ± SD (analysis of n = 5 randomly selected images) for 4–24 hpi (*p<0.05, ***p<0.001, ****p<0.0001, *****p<0.00001; two-tailed student’s t-test compared to HK-wt values).

Because several MA NS1 mutants altered NS1 protein localization in mouse cells at 16 hpi, we next assessed the pattern of cytoplasmic staining at 4, 8, 16 and 24 hpi to determine the temporal pattern of NS1 subcellular localization. We analyzed the NS1 mutants D125G and M106I+L98S, which exhibited the most dramatic cytoplasmic staining phenotype, as well as two prototype laboratory IAV strains that had been previously adapted to the mouse, A/WSN/1933 (H1N1) (WSN) and A/PR/8/1934 (H1N1) (PR8). Representative confocal images for each time point are shown (Figure 2a) and the proportion of antigen positive cells with cytoplasmic staining (for 5 images each of N = 15–70 cells/63× image) are shown for each time point in Figure 2b. At all time points, cells infected with HK-wt virus demonstrated a predominantly nuclear NS1 protein localization (Figure 2a), with an overall average of 12.4% of positively stained cells that demonstrated NS1 localized to the cytoplasm (Figure 2b). However, both PR8 and WSN viruses had a different NS1 localization phenotype, where each induced a significantly greater percentage of infected cells with NS1 detected in the cytoplasm at 4 h than HK-wt (33% and 28% versus 9.3%, respectively (p<0.01)) and again at 8 h (31.7% (p<0.05) and 84.3% (p<0.0001) versus 8.4%, respectively) but that were not significantly different from HK-wt at later time points (Figure 2b).

NS1 mutants M106I+L98S and D125G, like the PR8 and WSN viruses, also demonstrated an increase in cells positive for NS1 in the cytoplasm, however the kinetics of NS1 localization were delayed. Thus unlike PR8 and WSN, the rHK NS1 mutants showed a temporally progressive increase in cytoplasmic positive NS1 cells, with the greatest percentage at 24 hpi, where 85% or 65% of NS1 antigen positive cells stained for cytoplasmic NS1 respectively (p<0.00001 and p<0.0001, relative to HK-wt) (Figure 2b). These results indicated that the mouse-adaptive M106I+L98S and D125G NS1 mutations increased the prevalence of infected cells positive for cytoplasmic NS1 protein by a mechanism that increased with time following infection.

### Adaptive NS1 Mutants Increased the Relative Cytoplasmic to Nuclear Content of the NS1 Protein in Infected Mouse Cells

**Figure 3:**
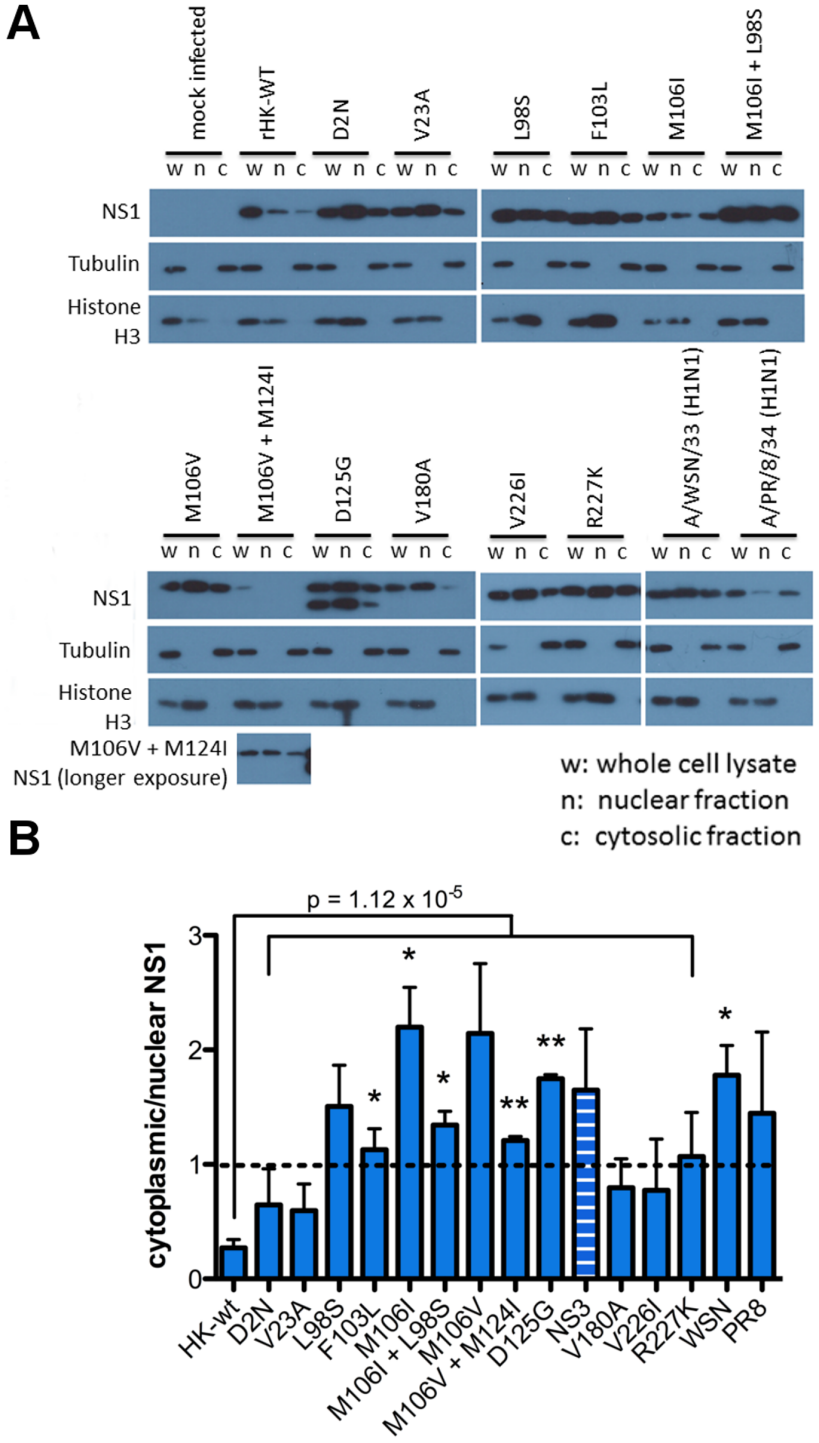
MA NS1 mutations alter subcellular localization of the NS1 protein in infected M1 cells. Mouse M1 cells were infected (MOI = 2) with rHK NS1 mutant viruses, the HK-wt virus, or the PR8, or WSN viruses, or mock infected with PBS. At 16 hpi the cells were lysed and differentially centrifuged to obtain whole cell (w), nuclear (n) and cytosolic (c) fractions. Cell fractions were resuspended in SDS buffer then were separated by SDS PAGE electrophoresis, followed by Western blot analysis to detect the NS1 protein as well as loading markers tubulin and histone H3. (A) Representative blot of 2–3 independent experiments, which were (B) analysed by densitometry where the amount of NS1 or NS3 protein in the nuclear and cytoplasmic fractions of a cell infected with a given virus was normalized to histone H3 and tubulin levels of the whole cell lysate, respectively. Cytoplasmic distribution was calculated by dividing cytoplasmic NS1 by nuclear NS1. Data represent the means ± SE (*p<0.05, **p<0.01; two-tailed student’s t-test).

We next quantified the effect of the adaptive NS1 mutations on altered subcellular distribution of the NS1 protein in infected mouse cells by Western blot of cellular fractions. M1 cells were infected with HK-wt and NS1 mutant viruses, as well as prototype PR8 and WSN viruses using a MOI = 2. Following 16 hpi, the cells were lysed with 1% NP-40 to acquire the whole cell fraction, or differentially centrifuged to obtain nuclear and cytoplasmic cellular fractions (n = 2–3), as per published protocols [23]. Figure 3a shows representative Western blots of cells infected with each virus (whole cell lysate (wc), nuclear fraction (n), and cytosolic (c) fraction) to detect NS1 protein as well as cytoplasmic (tubulin) and nuclear (histone H3) reference standards for 1 of 2 or 3 representative experiments. Densitometry was performed to quantify relative NS1 abundance in each cellular fraction, which was normalized to the abundance of the tubulin or histone H3 internal standards, respectively. We then determined the ratio of cytoplasmic to nuclear NS1 protein abundance (c/n NS1 index) by normalizing input levels to tubulin and histone H3 band intensities of whole cell fractions, respectively (Figure 3b; detailed in methods).

The HK-wt NS1 protein was found to be predominantly nuclear in mouse M1 cells (0.27 c/n NS1 index) (Figure 3b), which was in agreement with the confocal immunostaining of mouse cells infected with the HK-wt virus where cytoplasmic NS1 was detectable in the minority of antigen positive cells relative to strong nuclear staining (Figures 1–2). However, all NS1 mutants, as well as the prototype mouse-adapted strains WSN and PR8, resulted in a trend of >2-fold increase in the c/n NS1 index compared to the HK-wt virus (p = 1.12×10^5^, two-tailed t-test; Figures 3b). While the greatest proportion of NS1 localized to the cytoplasm was demonstrated by both mutations at aa site 106 (M106V (not statistically significant) and M106I (p<0.05)), cytoplasmic NS1 was reduced when expressed as double mutations, in combination with M124I and L98S, respectively (Figure 3b). These data indicated that an increased proportion of NS1 localized to the cytoplasm is a general phenotype among NS1 mouse-adapted mutants of human IAV. It is possible that the low proportion of human HK-wt virus NS1 protein in the cytoplasm of mouse cells may be a functional defect that is under selective pressure.

To control for equivalent levels of virus infection, we also probed for the viral nucleoprotein (NP) and matrix protein (M1), which corroborated previously published results [21], showing greater protein synthesis for all NS1 mutants(Figure S3a).

### Altered Human Influenza virus NS1 Localization Induced by Mouse-adapted Mutations is a Host Dependent Phenotype

**Figure 4:**
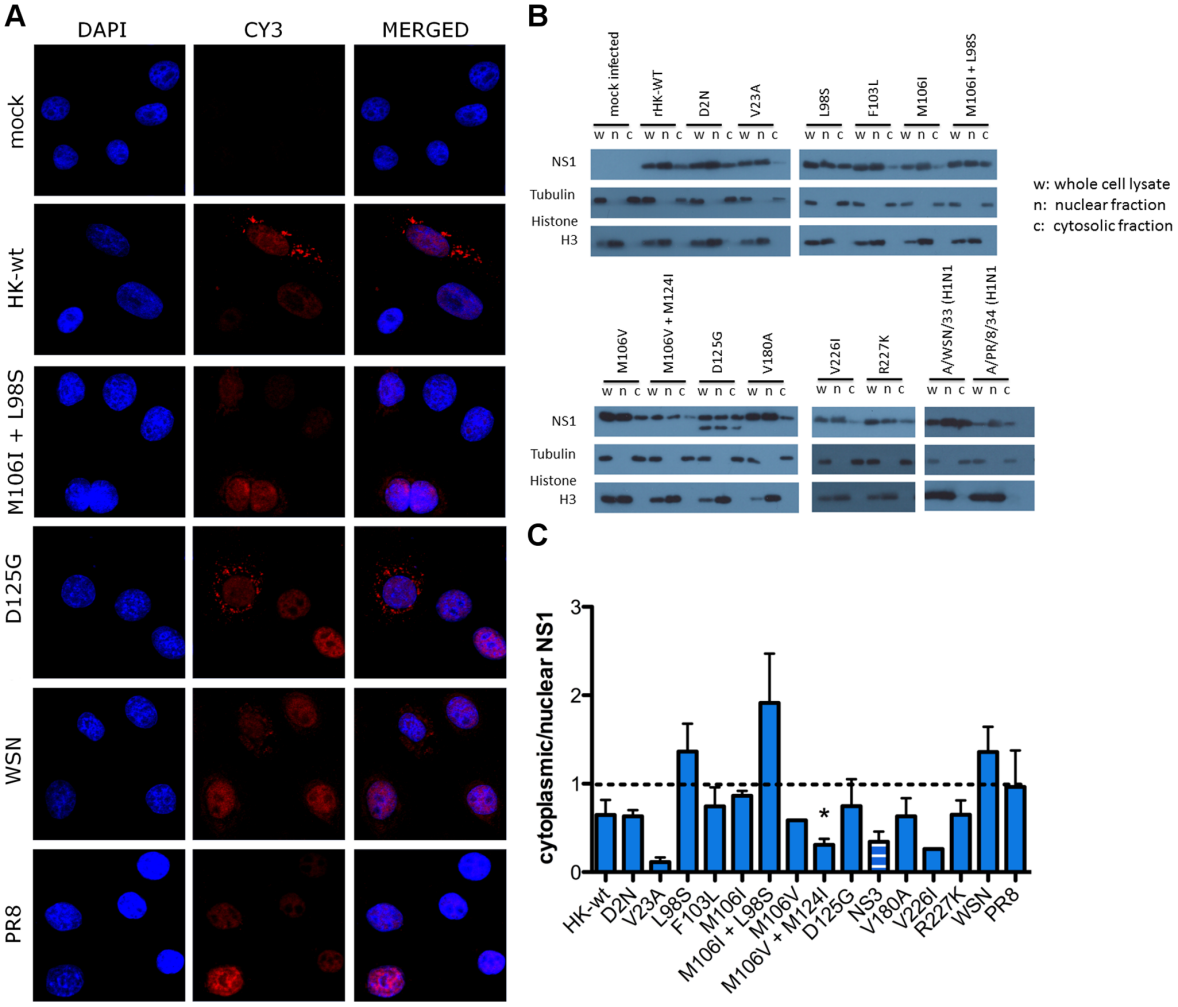
Increased cytoplasmic distribution of NS1 is a host-dependent phenotype of MA NS1 mutant viruses. (A) Human A549 cells were infected at MOI = 3 with the rHK NS mutant viruses, the HK-wt virus, or the WSN or PR8 viruses. Following 16 hpi, the cells were fixed and stained using a polyclonal anti-NS1 antibody and Cy3-conjugated secondary antibody as well as DAPI to localize the nucleus. Representative images are shown, taken at 63× using oil immersion. (B) Human A549 cells were infected with rHK NS mutant viruses, the HK-wt virus, or the WSN or PR8 viruses, or mock infected with PBS. At 16 hpi cells were lysed and differentially centrifuged to obtain whole cell (w), nuclear (n) and cytosolic (c) fractions. Cell fractions were resuspended in SDS buffer then were separated by SDS PAGE electrophoresis, followed by Western blot analysis to detect the NS1 protein as well as loading markers tubulin and histone H3. (B) Representative blot of 2–3 independent experiments, which were (C) analysed by densitometry where the amount of NS1 or NS3 protein in the nuclear and cytoplasmic fractions of a cell infected with a given virus was normalized to histone H3 and tubulin levels of the whole cell lysate, respectively. Cytoplasmic distribution was calculated by dividing cytoplasmic NS1 by nuclear NS1. Data represent the means ± SE (*p<0.05, two-tailed student’s t-test).

As an altered cellular distribution of the NS1 protein in infected mouse cells was observed for all MA NS1 mutants, we next asked whether this phenotype was host dependent. To this end, we assessed NS1 localization in infected human A549 cells by immunohistochemistry and confocal microscopy for NS1 mutants D125G and M106I+L98S, as well as the HK-wt, PR8 and WSN viruses. The cells infected with HK-wt displayed NS1 staining in both the nucleus and cytoplasm of A549 cells. This phenotype was also observed for cells infected with the aforementioned NS1 mutants as well as WSN (Figure 4a). However, cells infected with PR8 stained for NS1 primarily in the nucleus in human A549 cells (Figure 4a).

We then proceeded to quantitatively determine whether the distribution of the NS1 protein in infected A549 cells was altered by mouse-adapted NS1 mutations by cell fractionation and Western blot (Figures 4b, S3b). The HK-wt NS1 protein possessed a higher c/n NS1 index in infected human cells compared to infected mouse cells (0.64 versus 0.27; Figures 3b, 4c), and the majority of NS1 mutants (all but L98S and M106I+L98S) exhibited a similar or decreased proportion of cytoplasmic NS1 compared to HK-wt (Figure 4c). These observations suggest that altered NS1 localization is predominantly a host-dependent phenotype associated with IAV host adaptation, with the exception of the NS1 mutants possessing a mutation at L98S, which induced a higher proportion of NS1 localized to the cytoplasm in both cell lines (Figures 3 and 4).

### Increased Distribution of NS1 to the Cytoplasm is Associated with a Host-dependent Enhancement of viral Fitness

**Figure 5:**
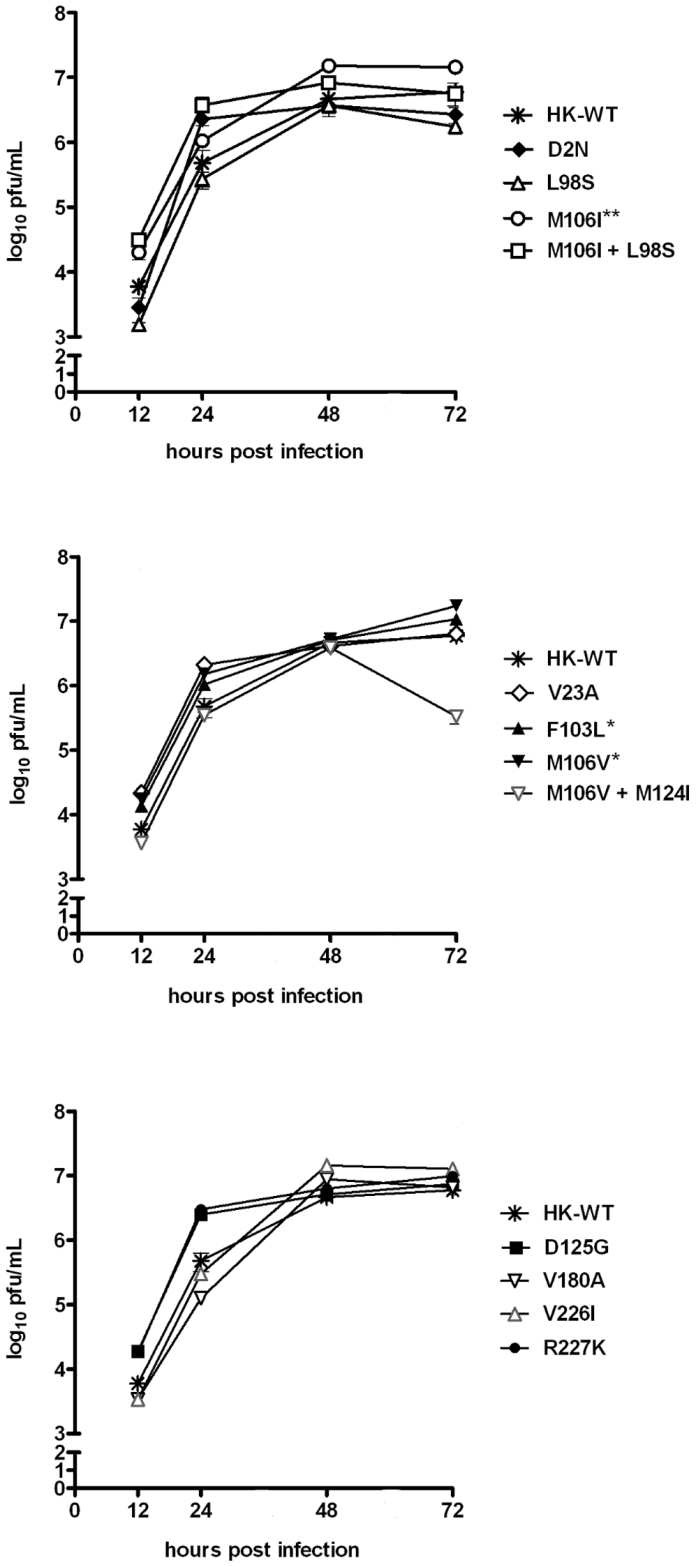
MA NS1 mutations enhance virus growth in human A549 cells in vitro. Human A549 cells were infected in triplicate at an MOI of 0.02 with rHK NS mutants or HK-wt virus, and supernatant collected 12, 24, 48, and 72 hpi was quantified for viral titre by plaque assay on MDCK cells. Data represent the mean viral titre of n = 3 replicates (each titrated in duplicate) ± SD (*p<0.05, **p<0.01, ***p<0.001; two-tailed student’s paired t-test for titers obtained from 12 to 72 hpi).

We have previously shown that MA NS1 mutations induced an average of a 21-fold increase in viral replication in mouse M1 cells compared to the HK-wt virus [21], and now report that these mutants tend to increase distribution of NS1 to the cytoplasm in mouse cells (Figure 3). We next explored whether the host-dependent alteration of NS1 localization associated with MA NS1 mutations correlated with viral replicative fitness by assessing virus replication in human A549 cells (n = 3). As the HK-wt virus was a human isolate, and presumably adapted to the human host, we hypothesized the virus would replicate to a higher level in human cells than in mouse cells. In respect to previously published virus growth curves in infected mouse cells, the HK-wt virus maximum yield was >1500 fold greater in human cells than in mouse cells (5.9×10^6^ pfu/mL versus 3.8×10^3^ pfu/mL [21], (Figure 5). This difference in host range for the HK-wt virus was consistent with diminished NP protein expression in human cells (Figure S3c).

The majority of viruses expressing NS1 mutations selected upon mouse adaptation were unable to increase virus replication in human cells, with 9 of the 12 mutants replicating to levels ≤ HK-wt (Figure 5). While NS1 mutants M106I, and to a lesser extent, F103L and M106V, did in fact increase virus replication throughout the time course (p<0.001, p<0.05, and p<0.05, respectively), the average level of enhancement among NS1 mutants amounted to only 1.5 fold; Figure 5). These results suggest that replicative fitness acquired upon adaptation to a novel host is largely a host-dependent phenotype for the NS1 mutations tested.

### Altered Host Gene Expression in Mouse Cells following IAV Infection due to Mouse Adapted NS1 Mutations

**Figure 6:**
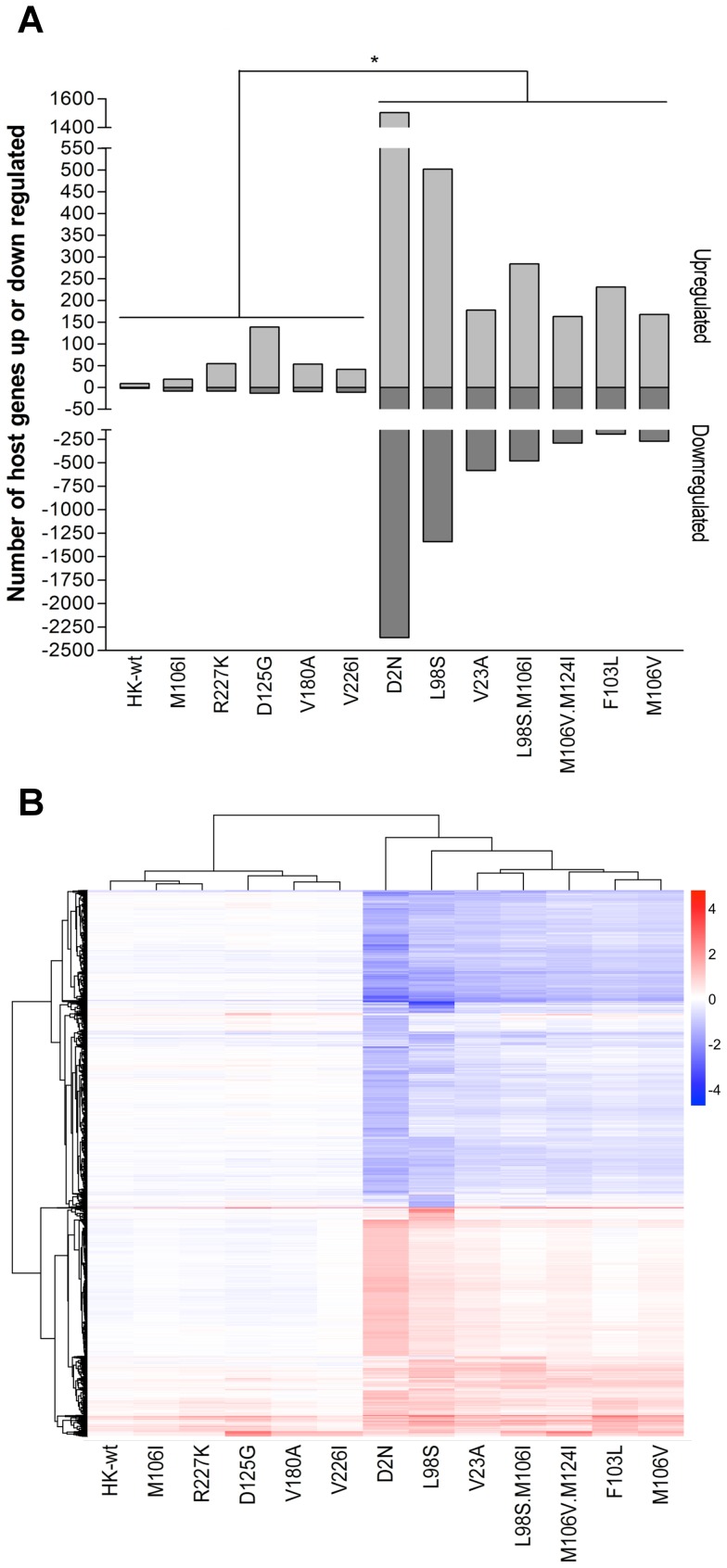
Mouse-adaptive NS1 mutations regulate mouse gene expression by two different mechanisms. Mouse M1 cells were infected in triplicate at an MOI of 2 with rHK NS mutants or HK-wt virus, and total RNA isolated at 8 hpi was analyzed by microarray using GeneChip Mouse Exon 1.0 ST Array (Affymetrix, Santa Clara, CA, USA). Microarray gene expression data were normalized and analyzed by Flexarray 1.6.1. Genes were considered as up or down regulated relative to mock infected cells if they were ≤1 or ≥1 log2 fold different (≤ or ≥2 fold differences) expression level (p≤0.05; ANOVA). (A) The number of differentially regulated host genes that were significantly up or down regulated ≤2 or ≥2 fold at the p≤0.05 by ANOVA is plotted for each mutant. The mutants formed two groups with either “low gene regulation” (LGR) or “high t gene regulation” (HGR) phenotypes. (B) Heat map of differentially regulated genes in mouse cells infected with HK-wt or rHK NS1 mutant viruses relative to mock infected M1 cells. Genes (total = 5274) were included for hierarchical clustering analysis among mutants if they were differentially regulated (≤2^−1^or ≥2^1^ fold differences) and significantly different from mock infected cells for one or more of the mutants, and gene regulation signatures were also analyzed for hierarchical clustering among viruses. The scale depicts up (red) and down (blue) regulated genes according to the log2 scale shown; equal expression is indicated in white (log 2^0^ = 1).

The NS1 protein has been shown to bind two cellular proteins involved in the post-transcriptional polyadenylation of host gene transcripts; nuclear poly-A binding protein II (PABPII), and cleavage and polyadenylation specificity factor 30, (CPSF30). The ability of NS1 to bind CPSF30 has been shown by transfection to positively correlate with reduced gene expression of target genes including IFN β [2], [26]. We have previously shown that MA NS1 mutations induced significant changes in binding affinity for CPSF30, with 8 mutants losing or reducing binding capacity [21]. However, there was no correlation between the ability to bind CPSF30 and the ability to shut off mouse IFN-β production *in vivo* [21]. We next wanted to determine whether the ability of the NS1 protein to bind CPSF30 was associated with a more general regulation of gene expression in mouse cells. To this end we performed microarray analyses using the mouse Affymetrix Genechip Mouse Exon 1.0 ST Array on RNA extracted from mouse cells 8 hours following infection with HK-wt and NS1 mutants at a MOI of 2. Three independent infections were analyzed in parallel with comparison of genes that were significantly up or down regulated by ≥1 or ≤1 log 2 fold (up or down regulated by ≥2.0 fold; p≤0.05 by ANOVA (n = 3)) relative to mock-infected M1 cells. On determining the host genes that were differentially regulated relative to mock infected cells, we observed that the viruses possessed one of 2 phenotypes, which had either low or high effects on host gene expression (Figure 6a; p≤0.05, number of genes 2 fold up- or down- regulated in expression compared to mock-infected cells). The low gene-regulating (LGR) group consisted of HK-wt and 5 of the 12 NS1 mutants (D125G, M106I, V180A, V226I, and R227K), where the number of genes up regulated exceed the number down regulated (p≤0.05), (average of 53 genes up regulated and 8.5 genes down regulated) (Figure 6a). Mouse cells infected with the 7 remaining MA NS1 mutants (D2N, V23A, F103L, M106I+L98S, L98S, M106V, and M106V+M124I) were found to activate more gene regulation events; compared to the LGR group of viruses, this high gene-regulating group (HGR) induced up-regulation and down-regulation of 8-fold and 93-fold more genes, respectively (p≤0.05) (Figure 6a).

**Figure 7:**
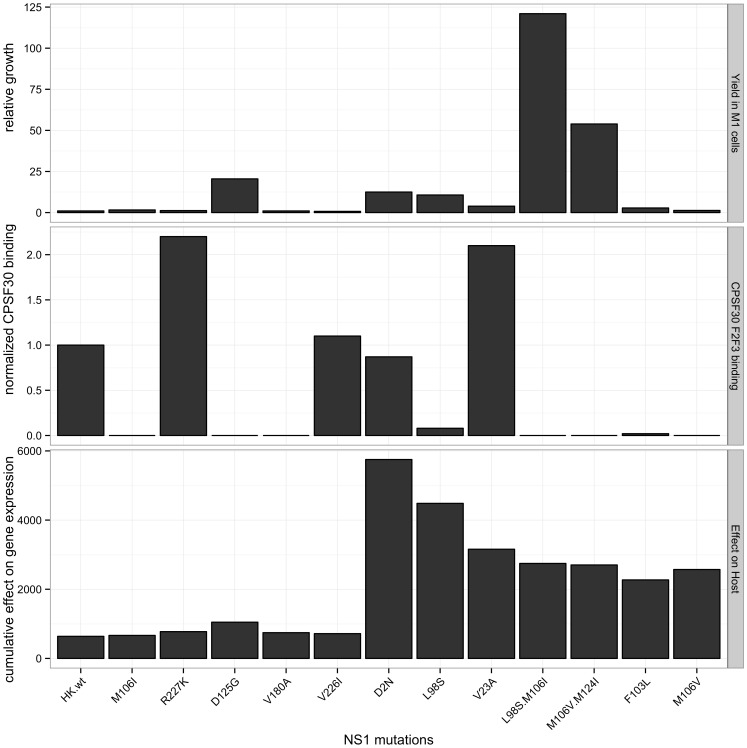
NS1 mutant CPSF30 binding affinity and ability to enhance viral replication in mouse cells does not correlate with regulation of mouse gene expression. Top panel: maximum virus yield in mouse M1 cells infected using a MOI of 0.02 relative to HK-wt virus yield (relative growth, top panel; values from [21]); middle panel: binding affinity to the CPSF30 F2F3-FLAG protein relative to HK-wt NS1 (normalized CPSF30 binding, middle panel; values from [21]), bottom panel: the cumulative effect on host gene expression of both up and down regulated genes as the sum of the absolute values of the log2 expression levels of host genes for each NS1 mutant.

We then performed hierarchical analyses of gene expression for the 5274 genes that were differentially regulated by at least one mutant. Gene regulation differences were illustrated upon histogram analysis of the HK-wt NS1 and each mutant to show the distribution of up and down regulated genes for each mutant (Figure S4). The six members of LGR group clustered together according to differential gene regulation. The M106I and R227K NS1 mutants most closely resembled HK-wt-induced host gene regulation, and differed from the closely linked V180A and V226I that were secondarily linked to the D125G mutant (Figure 6b). The hierarchical clustering of the HGR group showed 2 closely related clusters. NS1 mutant V23A clustered with M106I+L98S, and this hierarchical grouping was linked secondarily to a second cluster of mutants; F103L and M106V, which both previously were observed to induce similar host gene expression profiles in infected mouse cells (Figure 6b) [24]. The differentially regulated genes also fell into two main clusters of up and down regulated genes that were further divided into subgroups that were shared to different extents amongst the mutants (Figure 6b), indicating two distinct mechanisms of gene regulation for the NS1 mutants. Comparison of the gene regulation patterns (given as a relative statistic of the sum of the absolute values of the log2 fold differentially regulated genes for each virus) and virus properties of virus yields in M1 cells and CPSF binding [21], show a general lack of relationship among these parameters (Figure 7). Although the HGR group tended to generate higher yields of virus in mouse cells than the LGR group, both groups possessed high and low yielding viruses. These results suggest that the effects on host gene expression are not simply a measure of the extent of replication or ability to bind CPSF but are complex parameters (Figure 7).

Gene ontology (GO) analysis was performed at Level 2 for the average gene expression levels of the differentially regulated genes in the LGR and HGR groups. Although both groups included genes that were up and down regulated for host genes involved in biological processes, cellular components, and molecular functions, they differed fundamentally in the overall effect of these changes. The LGR group effects were dominated by positive regulation of host genes whereas the HGR group was dominated by negative regulation of genes suggesting that they provide adaptive gain of function through different mechanisms of host interaction (Figures S5, S6).

**Figure 8:**
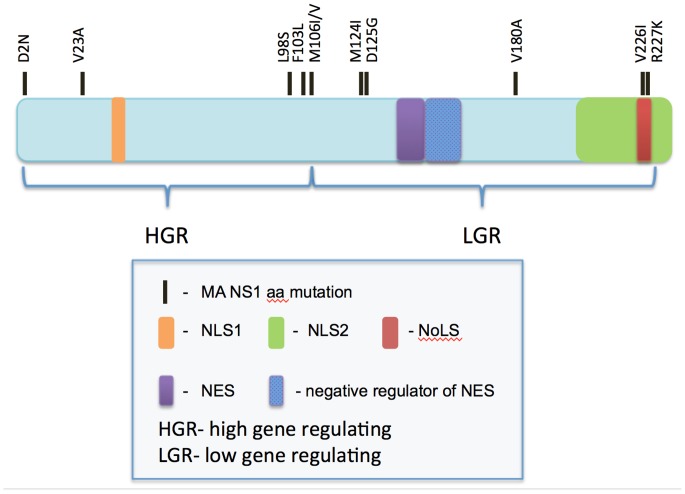
Protein map of NS1 indicating the location of mutations selected upon HK virus mouse adaptation. Linear map of NS1 protein indicating the location of NS1 mutations selected upon adaption of A/Hong Kong/1/1968 (H3N2) to the mouse, as depicted by black lines. NS1 nuclear localization sites (NLS1 and 2), nuclear export signal (NES), and its adjacent negative regulator of NES are indicated on the protein map as well as the amino and carboxyl protein regions associated with sites of mutations inducing high or low host gene regulation phenotypes (HGR or LGR, respectively).

The HGR and LGR mutations mapped to two regions of the NS1 protein, divided at aa position 106 (Figure 7 and 8). While these regions do not correspond with the accepted functional domain partitioning for the NS1 protein (RNA binding domain (aa 1–73) and effecter domain (aa 74–230) [1], our results do suggest different roles of the amino terminal versus carboxy-terminal domains of the NS1 gene in regulating host gene expression.

### NS1 Mutants Enhance Genomic viral RNA Replication in Infected Mouse Cells

**Figure 9:**
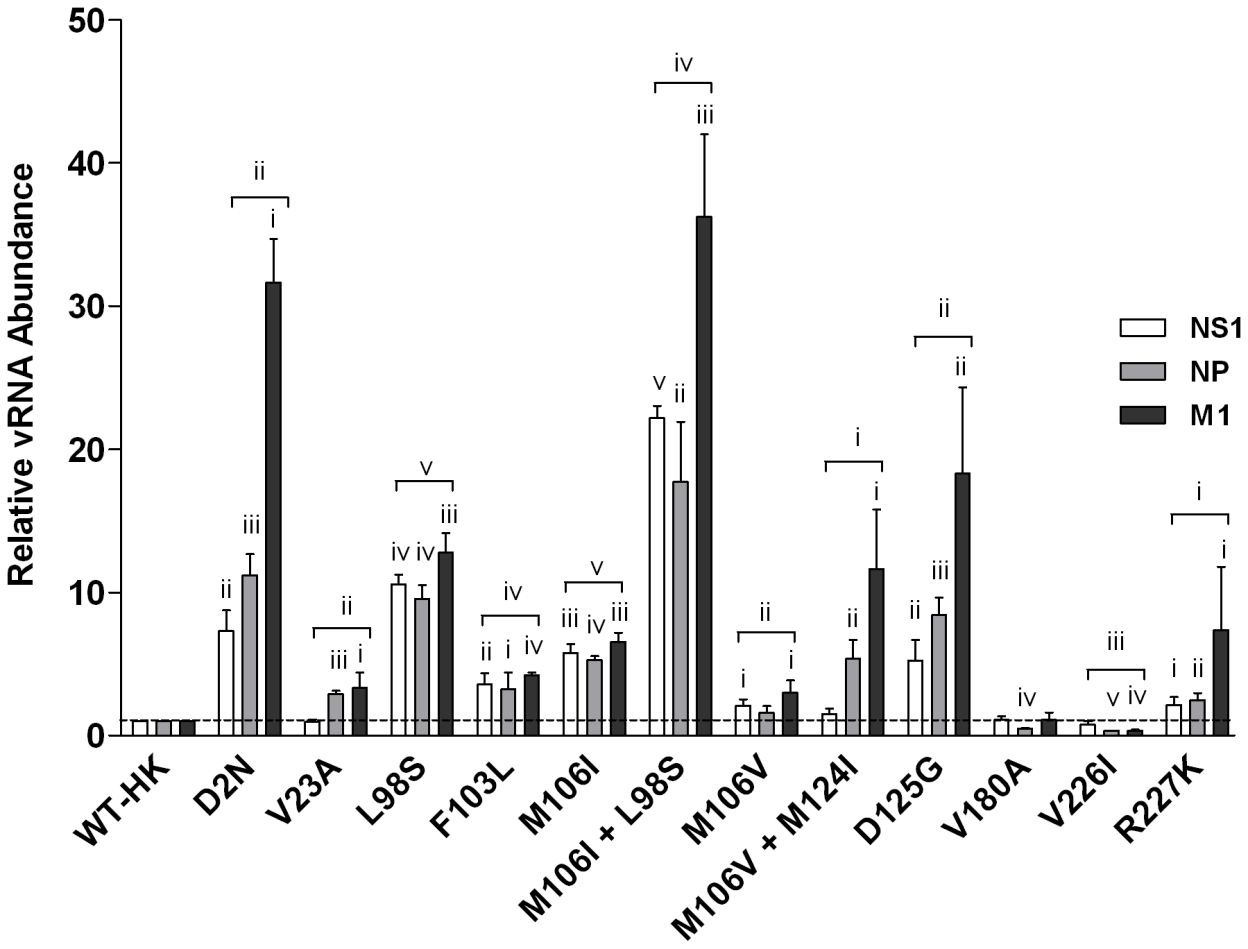
MA NS1 mutations increase genomic viral RNA synthesis in infected mouse cells. Mouse M1 cells were infected in triplicate at an MOI of 2 with rHK NS mutant viruses or the HK-wt virus, and total RNA isolated at 8 hpi was reverse transcribed using primers specific for genomic viral RNA segments. Real-time PCR (qPCR) was performed to quantify NP, M1 and NS1 genomic RNA levels. Results were normalized by β-actin levels, and presented as values relative to HK-wt genomic RNA levels. Data represent the means ± SD (two tailed student’s paired t-test) for NP, NS1 and M1 transcript relative levels (indicated by bracket) or two-tailed student’s t-test for individual mRNA samples (n = 3) relative values (i p<0.05; ii p<0.01; iii p<0.001; iv p<0.0001; v p<0.00001).

We have previously reported the majority of NS1 mutants significantly increase viral mRNA abundance in infected mouse cells [21]. We extended this analysis to determine the effects of the NS1 mutants on genomic viral RNA (vRNA) transcription. RNA extracts employed for microarray analysis were subjected to real-time quantitative RT-PCR analysis of genomic viral RNA (vRNA) encoding NS1, NP and M1 genes (n = 3 biological replicates with 2 technical replicates of each qPCR analysis). All NS1 mutants with the exception of V180A and V226I significantly increased the abundance of NS1, NP and M1 vRNA genes relative to HK-wt levels (p≤0.05, paired t-test; Figure 9). Comparing the fold differences relative to HK-wt levels, relative abundance of vRNA for the three tested transcripts were similar for V23A, L98S, F103L, M106I, M106V, V180A, and V226I, but differed for the remaining mutants (D2N, M106I+L98S, and R227K), that tended to possess higher relative abundances of the M1 vRNA relative to the NP and NS1 genome segments (Figure 9). These data demonstrated that the majority of NS1 mutations selected upon IAV adaptation to the mouse increase both viral transcription [21] and genome replication (Figure 9) but to different extents and different patterns among the three genes tested.

### Phenotypic Trends of Mouse Adapted NS1 Proteins

**Figure 10:**
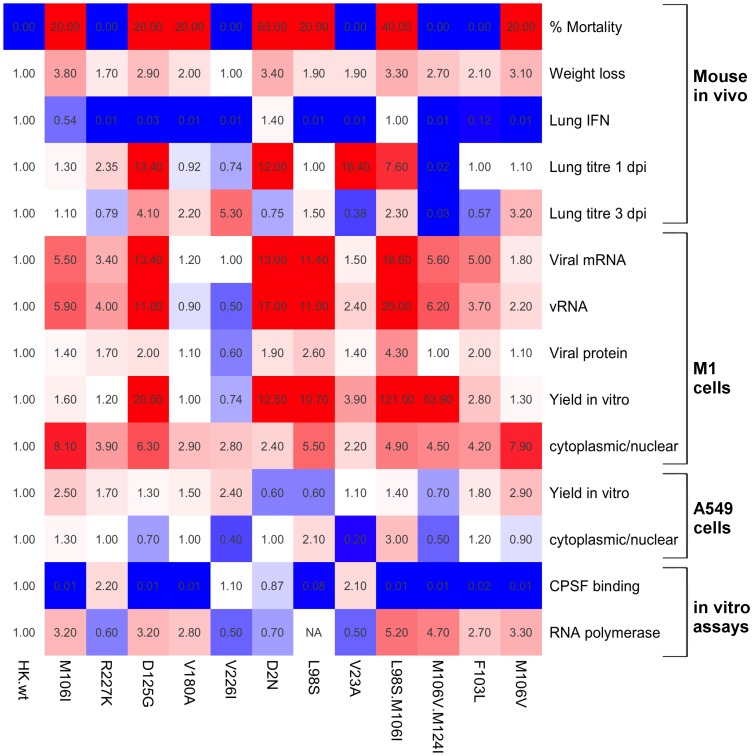
Adaptive properties of rHK viruses expressing NS1 mutations selected upon mouse adaptation. Heat map illustrating adaptive phenotypes (Figures 3–5, 7, 9, and [21]); relative to HK-wt values, where 1 is indicated as white* and the red scale depicts phenotypes greater than HK-wt (increasing shades of red from 1 to 10 (with saturated red for ≥10), and shades of blue scale from 1 to 0 depicts phenotypes lesser than HK-wt values, where data was calculated as a fold change relative to HK-wt value. For virus yield values, maximum virus yield obtained in the growth curve was used to calculate fold change relative to HK-wt virus maximum virus yield. *With the exception of % mortality in the mouse where 0% mortality is indicated as blue and any mutation inducing mortality in the mouse indicated as red.

Thus far we have shown several novel adaptive phenotypes associated with NS1 mutations selected on mouse adaptation, including altered NS1 protein localization, enhanced vRNA production, and regulation of host gene expression. Previous reports have indicated the ability to bind CPSF30 correlated with nuclear localization of the NS1 protein [12] as well as ability to shut down host gene expression by inhibiting pre-mRNA 3′ processing [27]. We tabulated the known phenotypic properties of the NS1 mutant panel to show that ability to bind the host factor CPSF did not correlate with intracellular localization of the NS1 protein (Figure 10). We therefore conclude additional mechanisms beyond CPSF binding are involved in host gene regulation by the NS1 gene.

## Discussion

We have previously shown that the NS1 mutations selected on IAV evolution in the mouse are adaptive, possessing multiple gain-of-function phenotypes including enhanced viral replication *in vivo* in the mouse lung and *in vitro* in mouse cells, enhanced viral gene expression at both the level of viral mRNA and viral protein in mouse cells, and ability to suppress IFN-β production in the mouse lung [21]. Moreover, observed reductions in IFN-β production *in vivo* did not correlate with increased binding to the cellular post-transcriptional processing factor CPSF30 [21] (Figure 10). In this paper, we expand the scope of phenotypes associated with MA NS1 mutations and demonstrate that host adaptation of IAV is associated with increased distribution of the NS1 protein into the cytoplasm of infected cells (Figures 3 and 4), and that this phenotype is host-dependent and thus restricted to the adapted host (mouse), and not the original host (human) (Figure 10). We also demonstrated that NS1 mutations selected upon mouse adaptation altered host gene expression profiles by one of two distinct but unknown mechanisms. Notably, gene ontology analysis showed that while the LGR phenotype was associated with positive regulation of genes involved in host processes, these host processes were negatively regulated for the HGR phenotype (Figures 6, S5, and S6).

### Altered NS1 Cellular Distribution is Associated with Mouse Adaptive Phenotypes of NS1 Mutations

The NS1 protein of the human prototype clinical isolate, HK-wt, was abundant in both the cytoplasm and nucleus of infected human A549 cells (Figure 4c) suggesting that this is an optimal distribution for a virus adapted to its host. Consistent with a defective ability to partition into the cytoplasm in the novel mouse host cell, HK-wt was predominately associated with the nucleus (Figure 3b). However all 12 mutant NS1 genes expressed in recombinant viruses in mouse cells showed a tendency to an increased proportion of NS1 protein in the cytoplasm versus the nucleus with statistically significant increases for the F103L, M106I, M106I+L98S, M106V+M124I, and D125G mutants (Figure 3). Prototype mouse-adapted laboratory strains PR8 and WSN expressed NS1 proteins that accumulated in the cytoplasm at levels ≥ nuclear NS1 levels for both mouse and human infected cells. This is possibly due to evolutionary changes selected on laboratory culturing as they both possess adaptive NS1 mutations 23A and 226I [21] and in addition PR8 possesses mutations 103S and 106I (both of which increase virulence) [24]. It is thus likely that host-specific cellular distribution of the NS1 protein contributed to the adaptive phenotypes associated with NS1 mutations. MA NS1 mutations enhance viral gene expression, which has previously been quantified at both the protein and mRNA, and now the vRNA, level in infected mouse cells (Figures 9 and 10) [21]. Increased NS1 gene expression accounts for greater cellular abundance of NS1 proteins; however the altered distribution to favour cytoplasmic localization suggests potential differences in nucleo-cytoplasmic transport or binding to host proteins, the extent to which remains unknown.

Following its synthesis by host translation machinery, nascent NS1 is found in the host cell cytoplasm, then rapidly localizes to the nucleus [5]. Most NS1 proteins, including the HK NS1, possess two NLS, NLS1 at aa 34–38 and NLS2 at aa 203–237 [28], which by interaction with importin-α [29], function in driving nuclear localization of NS1. The NS1 protein also contains one characterized NES (aa 138–147 FDRLETLILL) that may contribute to the cytoplasmic localization of the NS1 protein reported at later times following infection [6]. Regulation of nuclear export appears to be complex, as the adjacent region to the NES (aa 148–161) inhibits NES activity in transfected cells but not in infected cells [7]. Cellular localization of NS1 protein thus is a culmination of multiple counteracting activities such as NLS (1+2) versus NES (+regulator activities) that shuttle NS1 between the two cellular compartments, and this phenomena is also influenced by NS1 protein modifications or interactions with other viral proteins as described for several other viral proteins [30], [31].

In further support of our findings, NS1 cytoplasmic localization has also been mapped to aa 103 and 106 residues of the PR8 virus in MDCK cells [12], and the M101I residue mutation in 2003 HPAI H5N1 in infected A549 cells, that corresponds to the 106 position in the HK-wt virus [32]. NS1 aa residues 103L and 106I have also been linked to cytoplasmic localization of the H5N1 highly pathogenic avian influenza (HPAI) isolate A/HK/156/1997 NS1 protein in mouse cells, when expressed on the PR8 viral backbone [24]. With respect to these findings, it is possible the NS1 protein contains an additional domain that influences nuclear export or cytoplasmic retention spanning from aa 98 to 106. Additional experiments on the localization of NS1 and NS3 proteins by site directed mutagenesis of aa 98–106 or transference of this motif (LSEDWFMLM) to other proteins, is required to further define the functional roles of component aa in nuclear export and cytoplasmic localization. Nonetheless, the observation of adaptive mutations in the NS1 gene that affect subcellular distribution is consistent with the common theme of adaptive evolution of nuclear localization signals in RNA polymerase subunits that overcome defects in interacting with cellular trafficking proteins [15], [18], [33], [34].

### Functional Roles of Enhanced Expression and Subcellular Localization of the NS1 Protein

We have shown that NS1 adaptive mutations increase viral transcription and viral gene expression at the level of protein synthesis (Figures 3 and 10; [21]). Because NS1 functions in enhancing translation of viral mRNA [35], [36], mutants with increased accumulation of NS1 in the cytoplasm of infected mouse cells may further promote protein synthesis. Given the NS1 protein is more abundant in the cytoplasm of infected mouse cells for the majority of NS1 mutants compared to HK-wt NS1, cytoplasmic NS1 can more effectively promote initiation of viral protein synthesis by binding to viral mRNA and recruiting it to cellular translational factors or alternatively that NS1 mutants favourably affect binding affinity for translational factors such as eIF4GI and PABPI [35], [36]. Binding to cytoplasmic factors such as these could also contribute to sequestration of the NS1 protein into the cytoplasm. Nonetheless, altered host-dependent cellular localization of NS1 mutants could contribute positively to the enhancement of viral gene expression.

Interferon antagonism was another NS1 property enhanced by the majority of NS1 mutants (Figure 10, [21]), and likewise this adaptive phenotype may be partially attributed to altered cellular localization of the NS1 protein. In the cytoplasm of infected human cells, NS1 inhibits IFN production at the transcriptional level of RIG-I signalling, by directly binding RIG-I and multiple RIG-I activators including E3 ligase, TRIM25 [37], and dsRNA [38]–[40]. RNA binding as well as RIG-I and the E3 ligase Riplet binding [41] inhibit signaling and IFN activation in mouse cells. In a recent report by Dankar et al. (2013), NS1 residues 103L and 106I of the A/HK/156/1997 (H5N1) HPAI strain were found to increase NS1 localization to the cytoplasm of infected mouse cells, and also to mediate increased binding to RIG-I, which correlated with decreased IFN-β production in infected mouse cells [24]. Thus the increased distribution of NS1 to the cytoplasm observed for MA NS1 mutants may also contribute to inhibition of type I IFN production.

In addition, increased cytoplasmic NS1 may also contribute to an enhanced ability of NS1 to circumvent the IFN response. Cytoplasmic NS1 binds dsRNA, which sequesters it from activation of PKR and 2′5′-OAS [10], [42]–[44]. NS1 also inhibits dsRNA-independent PACT-mediated PKR activation [42]. Moreover, cytoplasmic NS1 is responsible for blocking STAT-1 signalling induced by type I IFN, which results in suppression of IFN-stimulated gene transcription [2]. We have previously shown that the majority of MA NS1 mutants shut down IFN production in vivo in infected mouse lungs (Figure 10) and suppress the effects of IFN on viral replication and viral protein synthesis in mouse cells [21]. However additional experiments including quantification of NS1 cellular distribution at earlier times following infection are required to support the hypothesis that increased distribution of NS1 to the cytoplasm increases NS1 IFN antagonistic properties.

### Alternative Mechanisms for NS1- mediated Host Gene Induction and Suppression

Gene ontology analysis of mouse genes regulated upon influenza virus expression indicates two general mechanisms of regulation of host gene expression that involve alternatively down- or up regulation of host gene expression that mapped to the amino or the carboxyl- regions spanning aa 2 to 106 and 106 to 227, respectively (Figures 6, 7, 8, 10 and S5–S6). This suggests that NS1 may be interacting with transcriptional regulators or factors in the nucleus and or cytoplasm, and that mutations selected upon mouse adaptation may be altering such interactions. Human H3N2 NS1 genes isolated since 1969 have been shown to possess a histone-H3 mimic sequence ARSK at position 226–229 that functions to competitively inhibit activation of host anti-viral mediator genes to enhance virus replication [45]. The 226–229 VRSK sequence in HK-wt NS1 was mutated in the V226I and R227K mutants raising the possibility that these mutations affect gene interactions through modification of this system; confirmation of which will require further analysis.

The NS1 protein has been reported to block host gene expression at the level of mRNA production by interaction with numerous host factors. In addition to binding CPSF30, NS1 also binds the polyA binding protein nuclear 1 (PABPN1) [46], as well as the host mRNA nucleocytoplasmic export machinery, NXF1/TAP [47], which leads to the inhibition and thus down regulation of nuclear export of processed mRNA. Further assays of protein and RNA interactions between NS1 mutants and other nuclear cellular binding factors would be necessary to determine whether altered affinity to host gene regulation factors is involved in the mechanism by which the NS1 protein alters host gene expression. Given the set of NS1 mutants tended toward one of two distinct gene regulating phenotypes, this suggests more than one NS1 gene-regulating mechanism is selected upon influenza virus adaptation to a novel host which may be further dependent on other adaptive mutations that are selected in other viral genes [1]–[4], [14].

In summary we have demonstrated a deficiency in cytoplasmic NS1 as a host barrier to infection with a human influenza virus in mouse cells that was overcome by adaptive mutations. Although there have been no previous reports on the roles of host adaptation in modulating NS1 subcellular localization, these findings demonstrate that host adaptation alters the ability of the NS1 protein to localize to the cytoplasm in infected cells, and this altered cellular distribution was associated with other adaptive phenotypes such as enhanced IFN antagonism and viral gene expression. Moreover, we have shown NS1 mutations selected upon mouse adaptation alter host gene regulation by different, but unknown, mechanisms irrespective of the ability to bind CPSF30.

## Supporting Information

Figure S1 **Infection with NS1 mutants results in detectable levels of NP production.** Mouse M1 cells were infected at MOI = 3 with rHK NS mutant or rHK-wt viruses as indicated. Following 16 hpi, cells were fixed and stained using a polyclonal anti-NS1 antibody and Cy3-conjugated secondary as well as DAPI to localize the nucleus. Representative images are shown, taken at 63× using oil immersion.
(TIF)

Figure S2 **Quantification of virus-infected mouse cells with detectable NS1 protein in the cytoplasm.** Mouse M1 cells were infected as described in figure 1. Data represent the average percentage of cells positive for the NS1 antigen detected in the cytoplasm ± SD (analysis of n = 5 randomly selected images) at 16 hpi (*p<0.05, ***p<0.001; two-tailed student’s t-test compared to HK-wt values).
(TIFF)

Figure S3 **Viral protein expression in mouse and human cells for rHK NS1 mutant or HK-wt viruses.** Cellular fractions of infected mouse cells (A; same samples as shown in figure 3) or infected human whole cell lysates (B; same samples as shown in figure 4) were separated by SDS page then probed with anti-NP and anti-M1 antibodies. (C) HK-wt virus produces less viral protein in infected mouse cells than in infected human cells (same samples as in figures 3 and 4). WC: whole cell lysate; N: nuclear fraction; C: cytoplasmic fraction; NP: nucleoprotein; M1: matrix protein 1.
(TIFF)

Figure S4 **Histogram analysis of the HK-wt NS1 and each mutant to show the distribution of up and down regulated genes.** The relative distribution of number of genes and their log2 regulation levels relative to mock infected M1 cells are shown for the genes analyzed by hierarchical clustering in Figure 6. The up and down regulated genes are show in shades of red and blue respectively according to the scale shown with values of 1 shown in white (2^0^ = 1).
(TIFF)

Figure S5 **Gene ontology (GO) analysis was performed at Level 2 for the average gene expression levels of the differentially regulated genes in the low host gene regulating (LGR) group.** The average gene expression levels among the set of 5,274 differentially regulated genes of the member of the LGR group of mutations (D125G, M106I, V180A, V226I, and R227K) were characterized for their up or down regulating effects for host genes involved in biological processes, cellular components, and molecular functions. The data show the number of genes that are either up or down regulated for each of the functional areas indicated. The LGR group effects were dominated by positive regulation of host genes.
(TIFF)

Figure S6 **Gene ontology (GO) analysis was performed at Level 2 for the average gene expression levels of the differentially regulated genes in the high host gene regulating (HGR) group.** The approach described for Fig S2 was done for the HGR group of mutants, (D2N, V23A, F103L, M106I+L98S, L98S, M106V, and M106V+M124I). The HGR group was dominated by negative regulation of genes.
(TIFF)

## References

[1] Hale BG, Randall RE, Ortin J, Jackson D (2008) The multifunctional NS1 protein of influenza A viruses. The Journal of general virology 89: 2359–2376. 18796704 10.1099/vir.0.2008/004606-0

[2] Kochs G, Garcia-Sastre A, Martinez-Sobrido L (2007) Multiple anti-interferon actions of the influenza A virus NS1 protein. Journal of virology 81: 7011–7021. 17442719 10.1128/JVI.02581-06 PMC1933316

[3] Noah DL, Twu KY, Krug RM (2003) Cellular antiviral responses against influenza A virus are countered at the posttranscriptional level by the viral NS1A protein via its binding to a cellular protein required for the 3′ end processing of cellular pre-mRNAS. Virology 307: 386–395. 12667806 10.1016/s0042-6822(02)00127-7

[4] Melen K, Kinnunen L, Fagerlund R, Ikonen N, Twu KY, et al (2007) Nuclear and nucleolar targeting of influenza A virus NS1 protein: striking differences between different virus subtypes. Journal of virology 81: 5995–6006. 17376915 10.1128/JVI.01714-06 PMC1900311

[5] Li Y, Lu X, Li J, Berube N, Giest KL, et al (2010) Genetically engineered, biarsenically labeled influenza virus allows visualization of viral NS1 protein in living cells. Journal of virology 84: 7204–7213. 20463066 10.1128/JVI.00203-10 PMC2898259

[6] Shaw MW, Compans RW (1978) Isolation and characterization of cytoplasmic inclusions from influenza A virus-infected cells. Journal of virology 25: 608–615. 625086 10.1128/jvi.25.2.608-615.1978 PMC353974

[7] Li Y, Yamakita Y, Krug RM (1998) Regulation of a nuclear export signal by an adjacent inhibitory sequence: the effector domain of the influenza virus NS1 protein. Proceedings of the National Academy of Sciences of the United States of America 95: 4864–4869. 9560194 10.1073/pnas.95.9.4864 PMC20179

[8] Melen K, Tynell J, Fagerlund R, Roussel P, Hernandez-Verdun D, et al (2012) Influenza A H3N2 subtype virus NS1 protein targets into the nucleus and binds primarily via its C-terminal NLS2/NoLS to nucleolin and fibrillarin. Virology journal 9: 167–422X-169-167. 22909121 10.1186/1743-422X-9-167 PMC3493336

[9] Donelan NR, Basler CF, Garcia-Sastre A (2003) A recombinant influenza A virus expressing an RNA-binding-defective NS1 protein induces high levels of beta interferon and is attenuated in mice. Journal of virology 77: 13257–13266. 14645582 10.1128/JVI.77.24.13257-13266.2003 PMC296096

[10] Min JY, Krug RM (2006) The primary function of RNA binding by the influenza A virus NS1 protein in infected cells: Inhibiting the 2′–5′ oligo (A) synthetase/RNase L pathway. Proceedings of the National Academy of Sciences of the United States of America 103: 7100–7105. 16627618 10.1073/pnas.0602184103 PMC1459024

[11] Li W, Noah JW, Noah DL (2011) Alanine substitutions within a linker region of the influenza A virus non-structural protein 1 alter its subcellular localization and attenuate virus replication. The Journal of general virology 92: 1832–1842. 21508188 10.1099/vir.0.031336-0 PMC3167881

[12] Han H, Cui ZQ, Wang W, Zhang ZP, Wei HP, et al (2010) New regulatory mechanisms for the intracellular localization and trafficking of influenza A virus NS1 protein revealed by comparative analysis of A/PR/8/34 and A/Sydney/5/97. The Journal of general virology 91: 2907–2917. 20826615 10.1099/vir.0.024943-0

[13] Volmer R, Mazel-Sanchez B, Volmer C, Soubies SM, Guerin JL (2010) Nucleolar localization of influenza A NS1: striking differences between mammalian and avian cells. Virology journal 7: 63. 20236536 10.1186/1743-422X-7-63 PMC2847567

[14] Brown EG, Bailly JE (1999) Genetic analysis of mouse-adapted influenza A virus identifies roles for the NA, PB1, and PB2 genes in virulence. Virus research 61: 63–76. 10426210 10.1016/s0168-1702(99)00027-1

[15] Brown EG, Liu H, Kit LC, Baird S, Nesrallah M (2001) Pattern of mutation in the genome of influenza A virus on adaptation to increased virulence in the mouse lung: identification of functional themes. Proceedings of the National Academy of Sciences of the United States of America 98: 6883–6888. 11371620 10.1073/pnas.111165798 PMC34447

[16] Ping J, Dankar SK, Forbes NE, Keleta L, Zhou Y, et al (2010) PB2 and hemagglutinin mutations are major determinants of host range and virulence in mouse-adapted influenza A virus. Journal of virology 84: 10606–10618. 20702632 10.1128/JVI.01187-10 PMC2950562

[17] Ping J, Keleta L, Forbes NE, Dankar S, Stecho W, et al (2011) Genomic and protein structural maps of adaptive evolution of human influenza A virus to increased virulence in the mouse. PloS one 6: e21740. 21738783 10.1371/journal.pone.0021740 PMC3128085

[18] Resa-Infante P GG (2013) The nuclear import machinery is a determinant of influenza virus host adaptation. Bioessays 35: 23–27. 23239226 10.1002/bies.201200138

[19] Keleta L, Ibricevic A, Bovin NV, Brody SL, Brown EG (2008) Experimental evolution of human influenza virus H3 hemagglutinin in the mouse lung identifies adaptive regions in HA1 and HA2. Journal of virology 82: 11599–11608. 18829764 10.1128/JVI.01393-08 PMC2583651

[20] Dankar SK, Wang S, Ping J, Forbes NE, Keleta L, et al (2011) Influenza A virus NS1 gene mutations F103L and M106I increase replication and virulence. Virology journal 8: 13. 21226922 10.1186/1743-422X-8-13 PMC3032709

[21] Forbes NE, Ping J, Dankar SK, Jia J-J, Selman M, et al (2012) Multifunctional Adaptive NS1 Mutations Are Selected upon Human Influenza Virus Evolution in the Mouse. PLoS ONE 7: e31839. 22363747 10.1371/journal.pone.0031839 PMC3283688

[22] Selman M, Dankar SK, Forbes NE, Jia J-J, Brown EG (2012) Adaptive mutation in influenza A virus non-structural gene is linked to host switching and induces a novel protein by alternative splicing. Emerging Microbes & Infections 1: e42. 26038410 10.1038/emi.2012.38 PMC3630925

[23] Suzuki K, Bose P, Leong-Quong RY, Fujita DJ, Riabowol K (2010) REAP: A two minute cell fractionation method. BMC research notes 3: 294. 21067583 10.1186/1756-0500-3-294 PMC2993727

[24] Dankar SK, Miranda E, Forbes NE, Pelchat M, Tavassoli A, et al (2013) Influenza A/Hong Kong/156/1997(H5N1) virus NS1 gene mutations F103L and M106I both increase IFN antagonism, virulence and cytoplasmic localization but differ in binding to RIG-I and CPSF30. Virol J 10: 243. 23886034 10.1186/1743-422X-10-243 PMC3733596

[25] M Blazejczyk MM, R Nadon (2007) FlexArray: A statistical data analysis software for gene expression microarrays. Montreal, Canada Genome Quebec.

[26] Kuo RL, Krug RM (2009) Influenza a virus polymerase is an integral component of the CPSF30-NS1A protein complex in infected cells. Journal of virology 83: 1611–1616. 19052083 10.1128/JVI.01491-08 PMC2643760

[27] Nemeroff ME, Barabino SM, Li Y, Keller W, Krug RM (1998) Influenza virus NS1 protein interacts with the cellular 30 kDa subunit of CPSF and inhibits 3′end formation of cellular pre-mRNAs. Molecular cell 1: 991–1000. 9651582 10.1016/s1097-2765(00)80099-4

[28] Greenspan D, Palese P, Krystal M (1988) Two nuclear location signals in the influenza virus NS1 nonstructural protein. Journal of virology 62: 3020–3026. 2969057 10.1128/jvi.62.8.3020-3026.1988 PMC253741

[29] Heikkinen LS, Kazlauskas A, Melen K, Wagner R, Ziegler T, et al (2008) Avian and 1918 Spanish influenza a virus NS1 proteins bind to Crk/CrkL Src homology 3 domains to activate host cell signaling. The Journal of biological chemistry 283: 5719–5727. 18165234 10.1074/jbc.M707195200

[30] Kosugi S HM, Tomita M, Yanagawa H (2008) Nuclear export signal consensus sequences defined using a localization-based yeast selection system. Traffic 9: 2053–2062. 18817528 10.1111/j.1600-0854.2008.00825.x

[31] Fulcher AJ JD (2011) Regulation of nucleocytoplasmic trafficking of viral proteins: an integral role in pathogenesis? Biochim Biophys Acta 1813: 2176–2190. 21530593 10.1016/j.bbamcr.2011.03.019 PMC7114211

[32] Meng J ZZ, Zheng Z, Liu Y, Wang H (2012) Methionine-101 from one strain of H5N1 NS1 protein determines its IFN-antagonizing ability and subcellular distribution pattern. Sci China Life Sci 55: 933–939. 23124793 10.1007/s11427-012-4393-9

[33] Gabriel G DB, Wolff T, Planz O, Klenk HD, Stech J (2005) The viral polymerase mediates adaptation of an avian influenza virus to a mammalian host. Proc Natl Acad Sci U S A 102: 18590–18595. 16339318 10.1073/pnas.0507415102 PMC1317936

[34] Gabriel G HA, Klenk HD (2008) Interaction of polymerase subunit PB2 and NP with importin alpha1 is a determinant of host range of influenza A virus. PLoS Pathogens. 10.1371/journal.ppat.0040011 PMC2222953 18248089

[35] Aragon T, de la Luna S, Novoa I, Carrasco L, Ortin J, et al (2000) Eukaryotic translation initiation factor 4GI is a cellular target for NS1 protein, a translational activator of influenza virus. Molecular and cellular biology 20: 6259–6268. 10938102 10.1128/mcb.20.17.6259-6268.2000 PMC86100

[36] Burgui I, Aragon T, Ortin J, Nieto A (2003) PABP1 and eIF4GI associate with influenza virus NS1 protein in viral mRNA translation initiation complexes. The Journal of general virology 84: 3263–3274. 14645908 10.1099/vir.0.19487-0

[37] Gack MU, Albrecht RA, Urano T, Inn KS, Huang IC, et al (2009) Influenza A virus NS1 targets the ubiquitin ligase TRIM25 to evade recognition by the host viral RNA sensor RIG-I. Cell host & microbe 5: 439–449. 19454348 10.1016/j.chom.2009.04.006 PMC2737813

[38] Guo Z, Chen LM, Zeng H, Gomez JA, Plowden J, et al (2007) NS1 protein of influenza A virus inhibits the function of intracytoplasmic pathogen sensor, RIG-I. American journal of respiratory cell and molecular biology 36: 263–269. 17053203 10.1165/rcmb.2006-0283RC

[39] Mibayashi M, Martinez-Sobrido L, Loo YM, Cardenas WB, Gale M Jr, et al (2007) Inhibition of retinoic acid-inducible gene I-mediated induction of beta interferon by the NS1 protein of influenza A virus. Journal of virology 81: 514–524. 17079289 10.1128/JVI.01265-06 PMC1797471

[40] Opitz B, Rejaibi A, Dauber B, Eckhard J, Vinzing M, et al (2007) IFNbeta induction by influenza A virus is mediated by RIG-I which is regulated by the viral NS1 protein. Cellular microbiology 9: 930–938. 17140406 10.1111/j.1462-5822.2006.00841.x

[41] Rajsbaum R AR, Wang MK, Maharaj NP, Versteeg GA, Nistal-Villan E, Garcia-Sastre A, Gack MU (2012) Species-specific inhibition of RIG-I ubiquitination and IFN induction by the influenza A virus NS1 protein. PLoS pathogens 8. 10.1371/journal.ppat.1003059 PMC3510253 23209422

[42] Li S, Min JY, Krug RM, Sen GC (2006) Binding of the influenza A virus NS1 protein to PKR mediates the inhibition of its activation by either PACT or double-stranded RNA. Virology 349: 13–21. 16466763 10.1016/j.virol.2006.01.005

[43] Min JY, Li S, Sen GC, Krug RM (2007) A site on the influenza A virus NS1 protein mediates both inhibition of PKR activation and temporal regulation of viral RNA synthesis. Virology 363: 236–243. 17320139 10.1016/j.virol.2007.01.038

[44] Tan SL, Katze MG (1998) Biochemical and genetic evidence for complex formation between the influenza A virus NS1 protein and the interferon-induced PKR protein kinase. Journal of interferon & cytokine research: the official journal of the International Society for Interferon and Cytokine Research 18: 757–766. 10.1089/jir.1998.18.757 9781815

[45] Marazzi I HJ, Kim J, Manicassamy B, Dewell S, Albrecht RA Seibert CW, Schaefer U, Jeffrey KL, Prinjha RK, Lee K, García-Sastre A, Roeder RG, Tarakhovsky A (2012) Suppression of the antiviral response by an influenza histone mimic. Nature 483: 428–433. 22419161 10.1038/nature10892 PMC3598589

[46] Chen Z, Li Y, Krug RM (1999) Influenza A virus NS1 protein targets poly(A)-binding protein II of the cellular 3′-end processing machinery. The EMBO journal 18: 2273–2283. 10205180 10.1093/emboj/18.8.2273 PMC1171310

[47] Satterly N, Tsai PL, van Deursen J, Nussenzveig DR, Wang Y, et al (2007) Influenza virus targets the mRNA export machinery and the nuclear pore complex. Proceedings of the National Academy of Sciences of the United States of America 104: 1853–1858. 17267598 10.1073/pnas.0610977104 PMC1794296

[48] Rahim MN, Selman M, Sauder PJ, Forbes NE, Stecho W, et al (2013) Generation and characterization of a new panel of broadly reactive anti-NS1 mAbs for detection of influenza A virus. J Gen Virol 94: 593–605. 23223621 10.1099/vir.0.046649-0

