# Supplementary figures and images for: Identification of Adaptive Mutations in the Influenza A Virus Non-Structural 1 Gene That Increase Cytoplasmic Localization and Differentially Regulate Host Gene Expression
Source: PLoS One. 2013 Dec 31;8(12):e84673. doi: 10.1371/journal.pone.0084673 (PMC3877356; doi:10.1371/journal.pone.0084673)

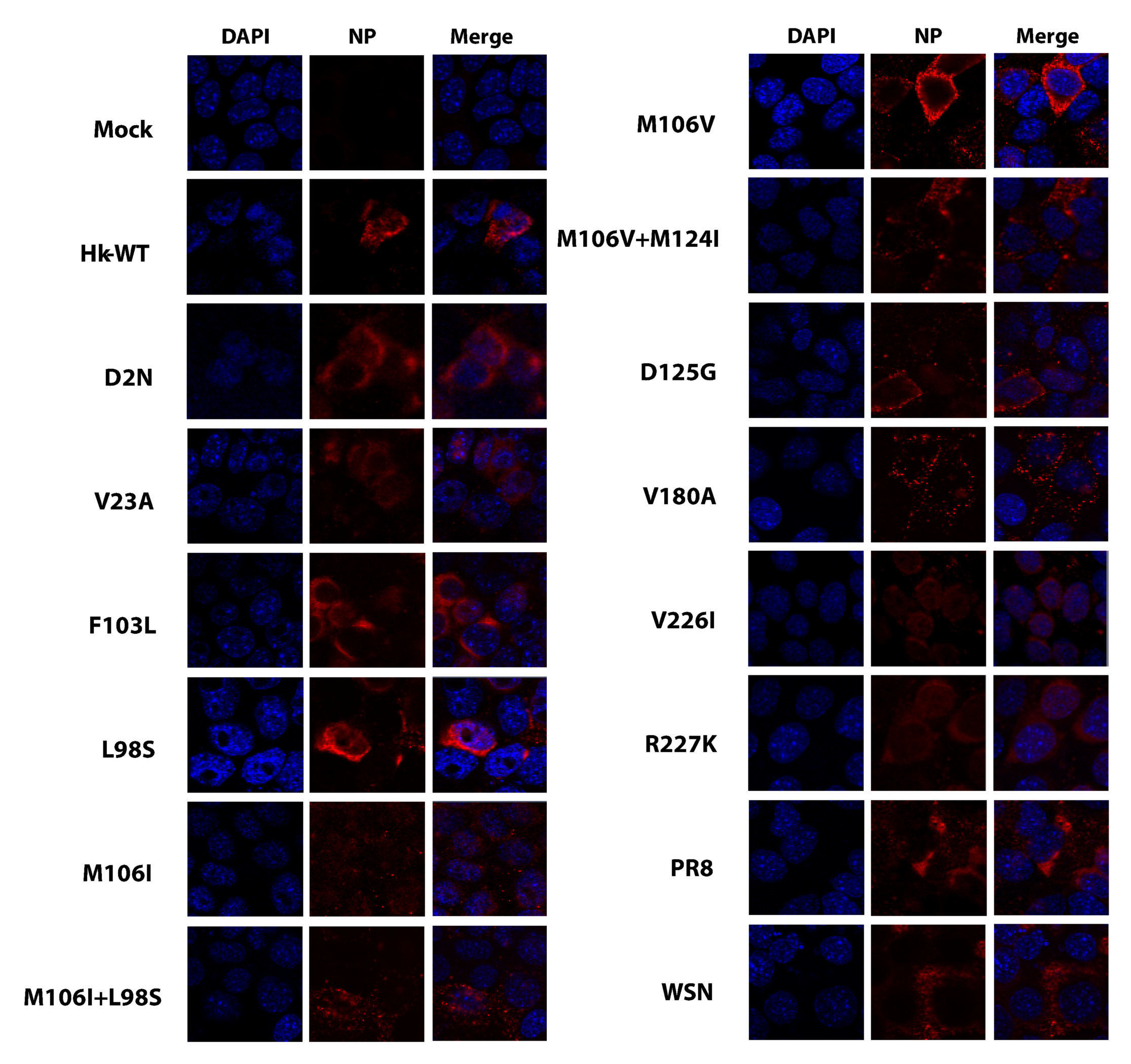

Supplement: Figure S1 — Infection with NS1 mutants results in detectable levels of NP production. Mouse M1 cells were infected at MOI = 3 with rHK NS mutant or rHK-wt viruses as indicated. Following 16 hpi, cells were fixed and stained using a polyclonal anti-NS1 antibody and Cy3-conjugated secondary as well as DAPI to localize the nucleus. Representative images are shown, taken at 63× using oil immersion. (TIF) [file pone.0084673.s001.tif]

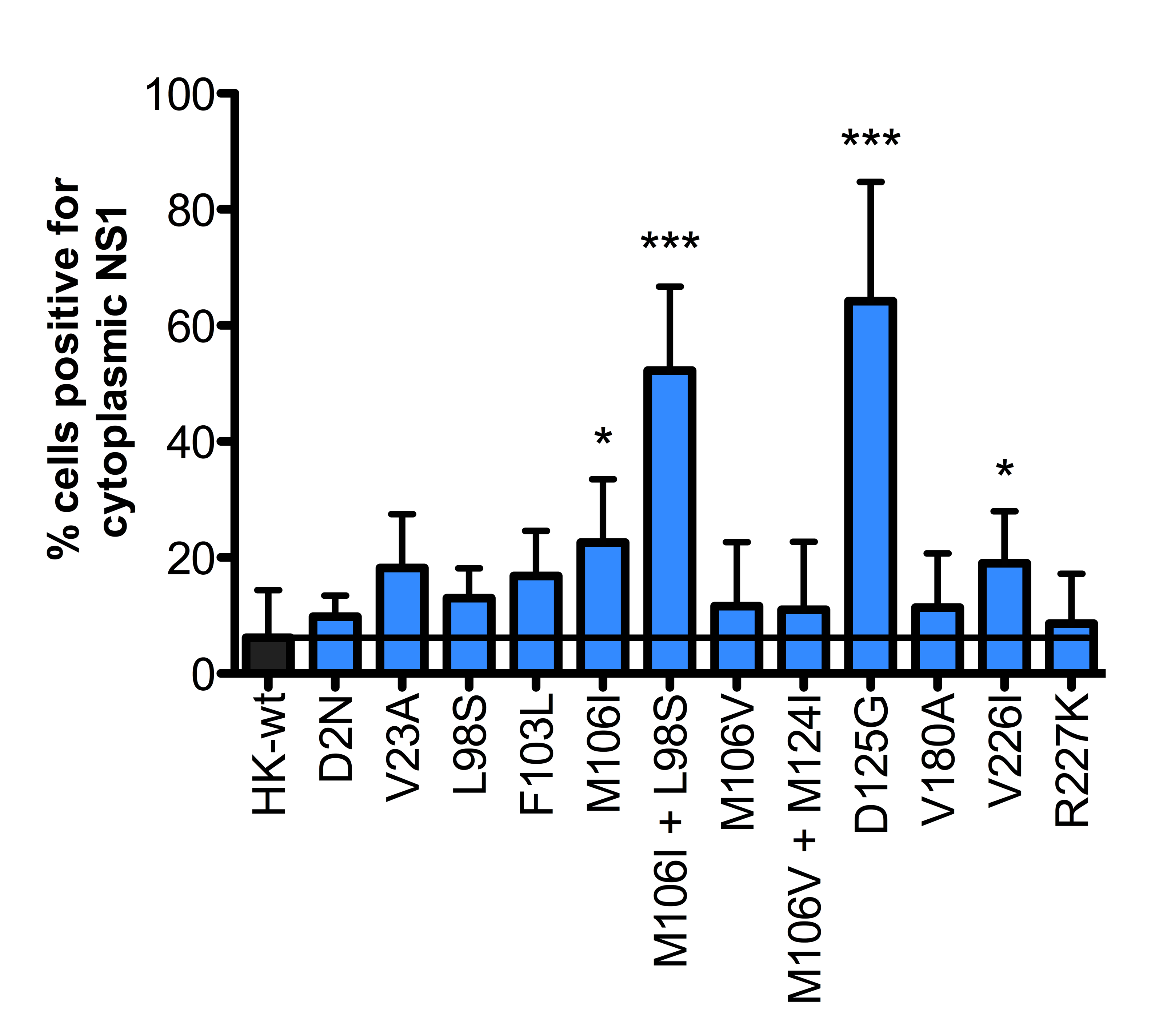

Supplement: Figure S2 — Quantification of virus-infected mouse cells with detectable NS1 protein in the cytoplasm. Mouse M1 cells were infected as described in figure 1. Data represent the average percentage of cells positive for the NS1 antigen detected in the cytoplasm ± SD (analysis of n = 5 randomly selected images) at 16 hpi (*p<0.05, ***p<0.001; two-tailed student’s t-test compared to HK-wt values). (TIFF) [file pone.0084673.s002.tiff]

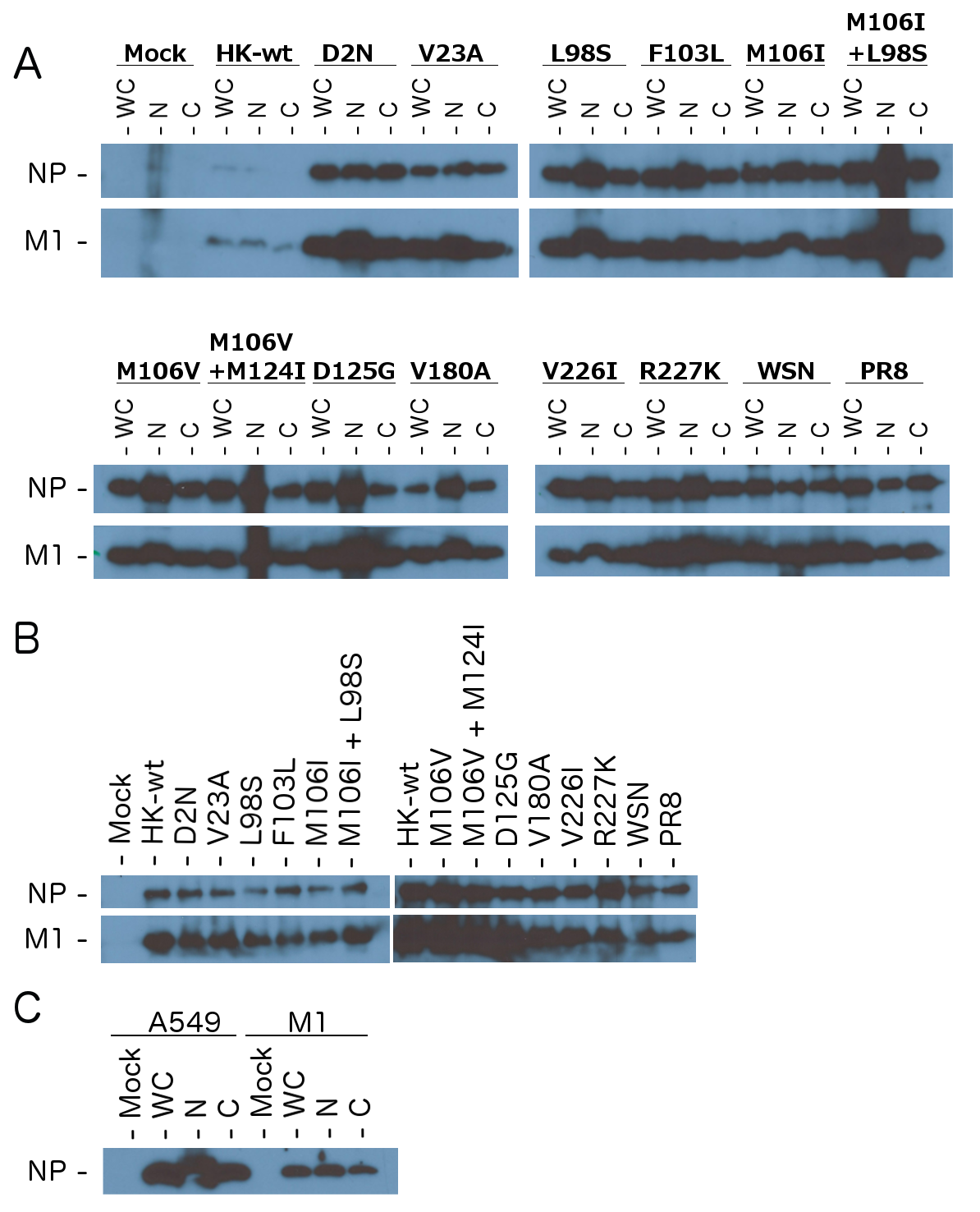

Supplement: Figure S3 — Viral protein expression in mouse and human cells for rHK NS1 mutant or HK-wt viruses. Cellular fractions of infected mouse cells (A; same samples as shown in figure 3) or infected human whole cell lysates (B; same samples as shown in figure 4) were separated by SDS page then probed with anti-NP and anti-M1 antibodies. (C) HK-wt virus produces less viral protein in infected mouse cells than in infected human cells (same samples as in figures 3 and 4). WC: whole cell lysate; N: nuclear fraction; C: cytoplasmic fraction; NP: nucleoprotein; M1: matrix protein 1. (TIFF) [file pone.0084673.s003.tiff]

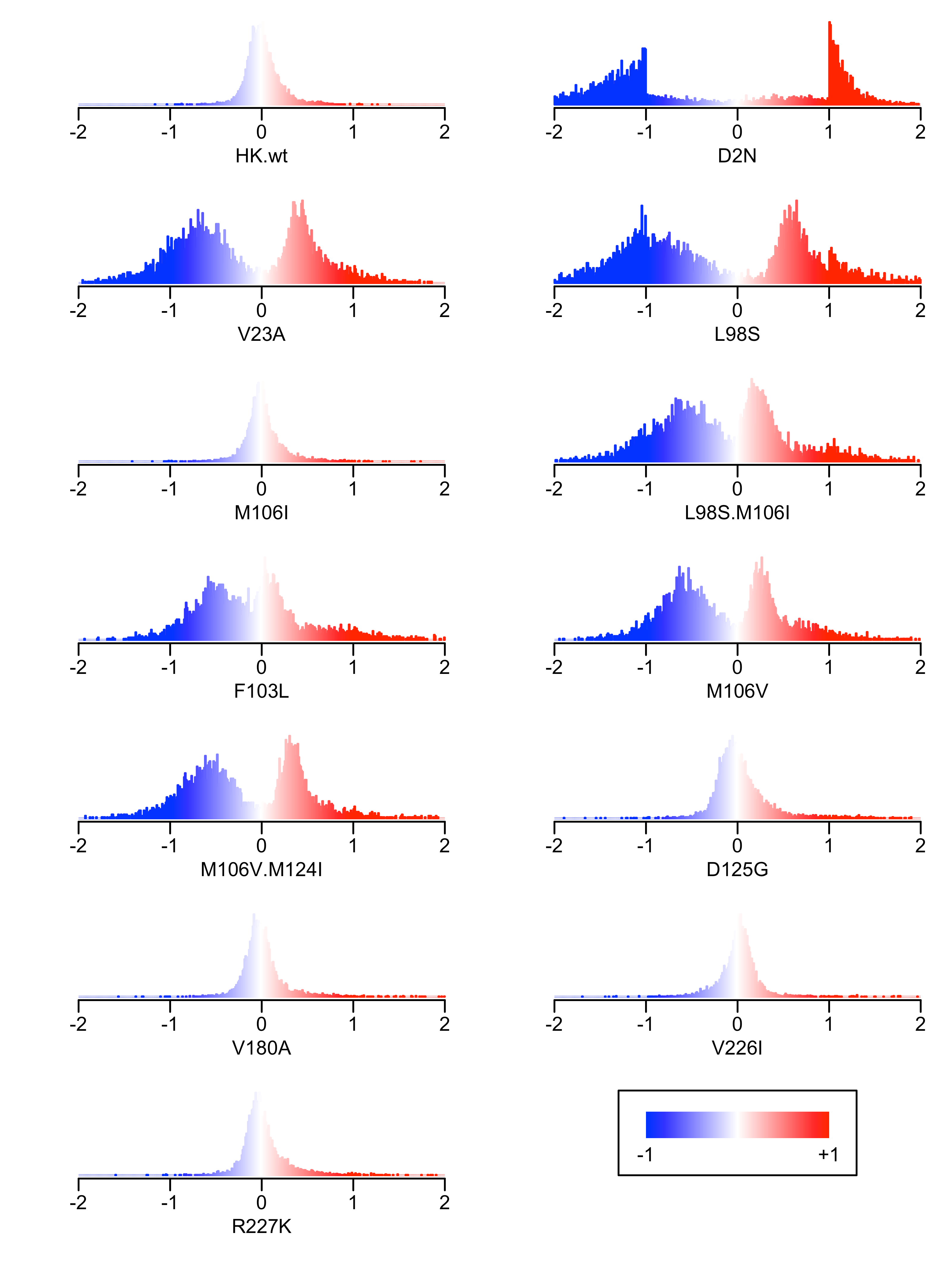

Supplement: Figure S4 — Histogram analysis of the HK-wt NS1 and each mutant to show the distribution of up and down regulated genes. The relative distribution of number of genes and their log2 regulation levels relative to mock infected M1 cells are shown for the genes analyzed by hierarchical clustering in Figure 6. The up and down regulated genes are show in shades of red and blue respectively according to the scale shown with values of 1 shown in white (20 = 1). (TIFF) [file pone.0084673.s004.tiff]

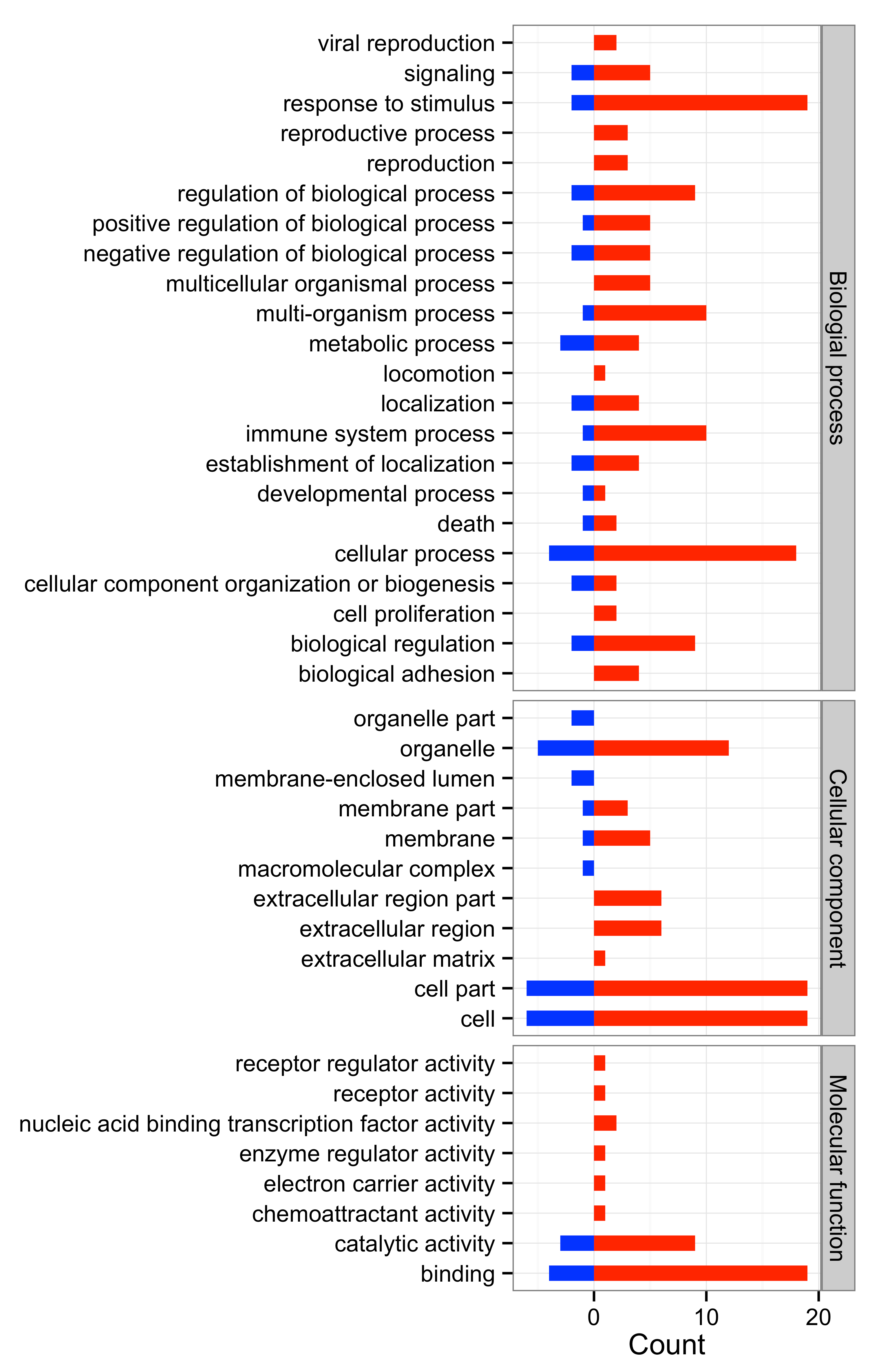

Supplement: Figure S5 — Gene ontology (GO) analysis was performed at Level 2 for the average gene expression levels of the differentially regulated genes in the low host gene regulating (LGR) group. The average gene expression levels among the set of 5,274 differentially regulated genes of the member of the LGR group of mutations (D125G, M106I, V180A, V226I, and R227K) were characterized for their up or down regulating effects for host genes involved in biological processes, cellular components, and molecular functions. The data show the number of genes that are either up or down regulated for each of the functional areas indicated. The LGR group effects were dominated by positive regulation of host genes. (TIFF) [file pone.0084673.s005.tiff]

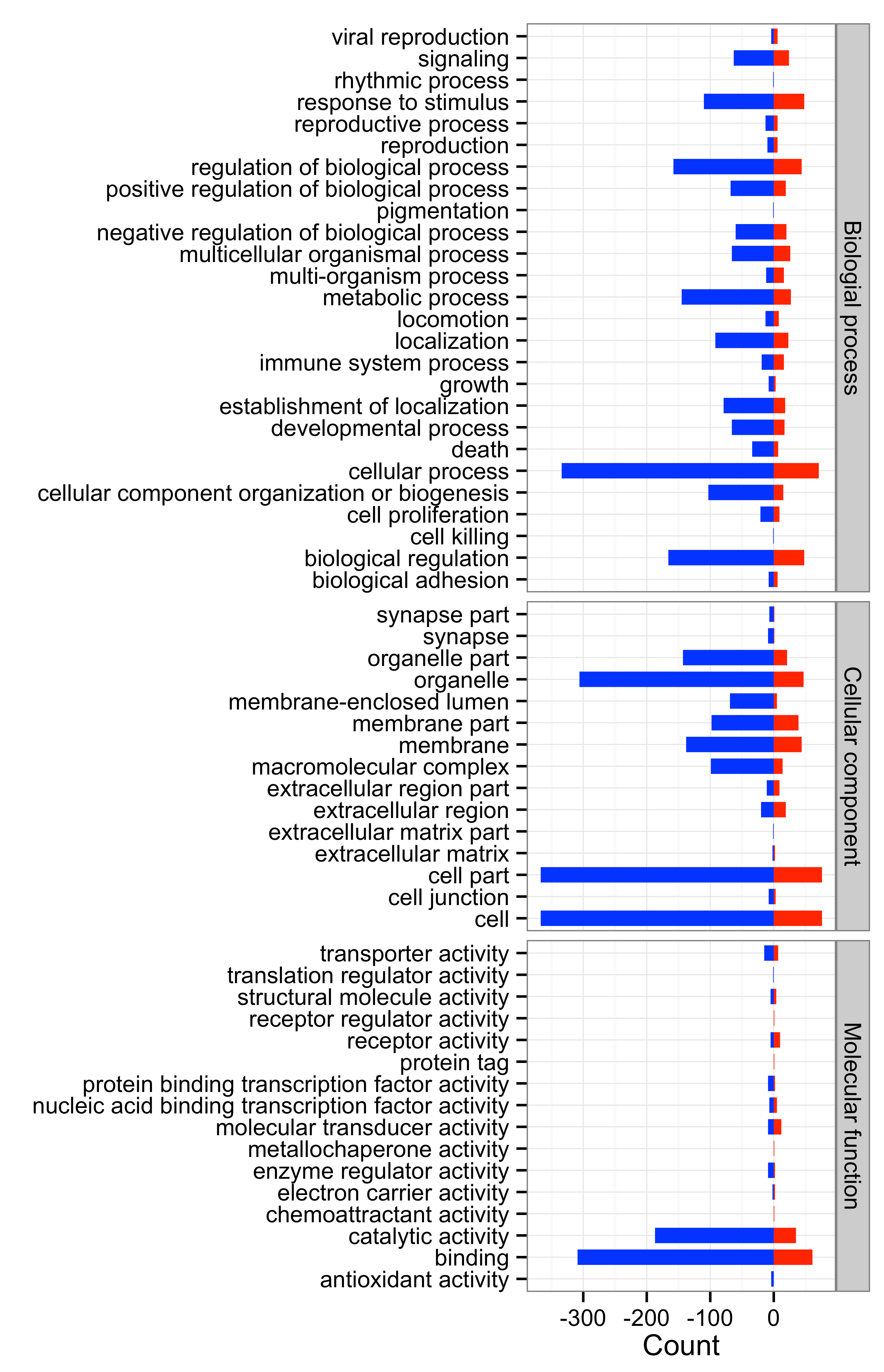

Supplement: Figure S6 — Gene ontology (GO) analysis was performed at Level 2 for the average gene expression levels of the differentially regulated genes in the high host gene regulating (HGR) group. The approach described for Fig S2 was done for the HGR group of mutants, (D2N, V23A, F103L, M106I+L98S, L98S, M106V, and M106V+M124I). The HGR group was dominated by negative regulation of genes. (TIFF) [file pone.0084673.s006.tiff]
